# Transcriptional profiling of the human fibrillin/LTBP gene family, key regulators of mesenchymal cell functions

**DOI:** 10.1016/j.ymgme.2013.12.006

**Published:** 2014-05

**Authors:** Margaret R. Davis, Robin Andersson, Jessica Severin, Michiel de Hoon, Nicolas Bertin, J. Kenneth Baillie, Hideya Kawaji, Albin Sandelin, Alistair R.R. Forrest, Kim M. Summers, for the FANTOM Consortium

**Affiliations:** a The Roslin Institute and Royal (Dick) School of Veterinary Studies, University of Edinburgh, Easter Bush EH25 9RG, UK; b The Bioinformatics Centre, Department of Biology and Biotech Research and Innovation Centre, University of Copenhagen, Ole Maaloes Vej 5, 2200 Copenhagen N, Denmark; c RIKEN Omics Science Center, Yokohama, Kanagawa 230-0045, Japan^1^; d RIKEN Center for Life Science Technologies, Division of Genomic Technologies, Yokohama, Kanagawa 230-0045, Japan; e RIKEN Preventive Medicine and Diagnosis Innovation Program, Wako, Saitama 351-0198, Japan; f The University of Queensland Northside Clinical School, Prince Charles Hospital, Chermside 4032, Australia

**Keywords:** FANTOM, Functional Annotation of Mammals, CAGE, cap analysis of gene expression, ECM, extracellular matrix, TB domain, latent transforming growth factor β binding domain, Fibrillin, Latent transforming growth factor β binding protein, Transcription start sites, Gene regulation, Extracellular matrix, Promoter

## Abstract

The fibrillins and latent transforming growth factor binding proteins (LTBPs) form a superfamily of extracellular matrix (ECM) proteins characterized by the presence of a unique domain, the 8-cysteine transforming growth factor beta (TGFβ) binding domain. These proteins are involved in the structure of the extracellular matrix and controlling the bioavailability of TGFβ family members. Genes encoding these proteins show differential expression in mesenchymal cell types which synthesize the extracellular matrix. We have investigated the promoter regions of the seven gene family members using the FANTOM5 CAGE database for human. While the protein and nucleotide sequences show considerable sequence similarity, the promoter regions were quite diverse. Most genes had a single predominant transcription start site region but *LTBP1* and *LTBP4* had two regions initiating different transcripts. Most of the family members were expressed in a range of mesenchymal and other cell types, often associated with use of alternative promoters or transcription start sites within a promoter in different cell types. *FBN3* was the lowest expressed gene, and was found only in embryonic and fetal tissues. The different promoters for one gene were more similar to each other in expression than to promoters of the other family members. Notably expression of all 22 *LTBP2* promoters was tightly correlated and quite distinct from all other family members. We located candidate enhancer regions likely to be involved in expression of the genes. Each gene was associated with a unique subset of transcription factors across multiple promoters although several motifs including MAZ, SP1, GTF2I and KLF4 showed overrepresentation across the gene family. *FBN1* and *FBN2*, which had similar expression patterns, were regulated by different transcription factors. This study highlights the role of alternative transcription start sites in regulating the tissue specificity of closely related genes and suggests that this important class of extracellular matrix proteins is subject to subtle regulatory variations that explain the differential roles of members of this gene family.

## Highlights

• We examine expression, promoter use and enhancers for the fibrillin/LTBP gene family.
• Promoter switching was observed for most family members.
• Multiple enhancers were identified for all family members.
• Family members overlapped in tissue specificity with some unique expression patterns.
• A degree of redundancy among family members is possible.

## 1 Introduction

Vertebrate genomes contain many gene families, consisting of genes with related sequence that arose through a range of duplication events (reviewed by [1]). The existence of gene families can provide redundancy and resilience to the genome, since one family member may substitute (at least in part) for another which has been mutated. This has been shown in knockout mouse models where a severe phenotype may only be found when several family members are inactivated. For example, several members of the SRC gene family had to be deleted to produce a myeloid phenotype (reviewed by [2]). The presence of multiple ion channel genes in the human genome led to the concept of repolarization reserve: since there are multiple channels capable of transporting potassium, sodium and calcium ions in cardiac myocytes, the heart beat can be maintained even in the presence of one non-functional protein [3,4]. Thus mice homozygous for a deletion of the potassium channel gene *Kcnd2* had no physiological or clinical phenotype, attributed to upregulation of potassium ion channels encoded by the *Kcna4* and *Kcna5* genes [5]. The possibility that members of a gene family may be able to substitute for each other has implications for genetically determined clinical conditions. To assess overlapping roles it is important to understand the structural and functional relationships between gene family members. In this study we have used the FANTOM5 promoter-based expression atlas encompassing the large majority of human cell types to examine promoter architecture and expression of members of the human fibrillin/LTBP gene family.

**Fig. 1:**
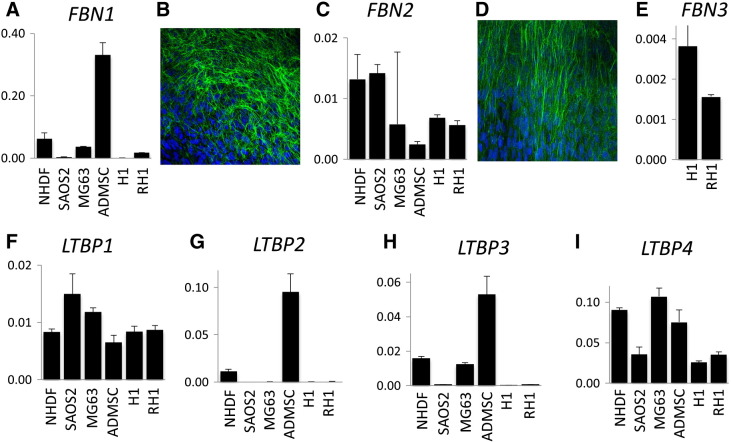
Expression of fibrillin/LTBP gene family members. qPCR was performed on RNA from cell lines SAOS2, MG63, H1 human embryonic stem cells and RH1 human embryonic stem cells and from primary cells ADMSC and NHDF. Results were normalized using *GAPDH*. *FBN3* was detected solely in H1 and RH1 embryonic stem cells. See Methods for more details. (A. *FBN1*; C. *FBN2*; E. *FBN3*; F. *LTBP1*; G. *LTBP2*; H. *LTBP3*; I. *LTBP4*). Fluorescent immunocytochemistry was performed on Day 7 cultured human fibroblasts, NHDF. Antibody information is available in Supplementary Table 1. (B. fibrillin-1; D. fibrillin-2).

The fibrillins and latent transforming growth factor binding proteins (LTBPs) form a small family of extracellular matrix (ECM) proteins characterized by the presence of a unique domain, the transforming growth factor beta (TGFβ) binding domain (TB domain) [6]. These proteins consist primarily of repeated epidermal growth factor (EGF) domains, most with the ability to bind calcium (Ca-EGF domains), interspersed with TB domains (reviewed by [7]; see Fig. 1 of that paper). The family members are important to both the structural integrity of the ECM and the regulation of bioavailability of members of the TGFβ family of growth factors. As well as being structurally similar, fibrillins and LTBPs appear to interact functionally in the sequestering and hence inactivation of TGFβ family members [8].

In vertebrates, including eutherian, marsupial and monotreme mammals, birds, reptiles and fish, fibrillins are encoded by three genes, *FBN1*, *FBN2* and *FBN3*. In rats and mice the *FBN3* gene appears to have degenerated and does not produce a functional mRNA [9], but in most mammals *FBN3* is likely to be active since transcripts have been detected (data from http://www.ensembl.org). There are a variable number of annotated LTBP genes across species, from two in fish to four in mammals: *LTBP1*, *LTBP2*, *LTBP3* and *LTBP4*. It is possible that one or more of the gene family members take over the role of *FBN3* in rats and mice.

Expression of fibrillin/LTBP family members is principally found in cells and tissues of mesenchymal origin. In mouse, *Fbn1* mRNA is ubiquitous in mesenchymal cell types [10], whereas *Fbn2* appears more restricted in expression ([7]; see http://biogps.org, data for *Fbn2*). A similar pattern was reported for human *FBN2* [7]. Human *FBN3* expression is restricted to embryonic/fetal tissues [9]. The LTBPs are also expressed primarily in cell types of mesenchymal origin, particularly osteoblasts and chondrocytes (http://biogps.org; [7]). This restricted expression suggests that there may be common regulatory elements, permissive for expression in mesenchymal cells, in the promoter regions of the seven genes, with specific elements determining the precise cell types in which the gene is expressed.

Consistent with their function in mesenchymal cell types, mutations in members of this gene superfamily result in phenotypes that primarily affect connective tissue types (reviewed in [7]). Although some aspects of the phenotypes overlap, each gene is associated with a unique spectrum of anomalies, reflecting the cell/tissue specific expression pattern [7]. Understanding the relationships between the family members and their differential regulation may lead to novel therapies in which alternative genes are upregulated to compensate for the mutated gene (as has been suggested for treatment of Duchenne muscular dystrophy by upregulating the dystrophin paralogue utrophin [11]).

The FANTOM (Functional Annotation of Mammals) projects co-ordinated by the RIKEN Institute in Japan have provided extensive information on gene expression in human and mouse and allowed the identification and characterization of a large number of gene promoter regions [12,13], using cap-selected 5′ end sequencing of mRNAs (cap analysis of gene expression; CAGE). Promoters were described as being sharp (with transcription initiating on a single base, usually associated with a conventional TATA box) or broad (with transcription initiating over a range of bases, usually associated with a CpG island). Analysis of transcription start sites also showed that genes often have alternative promoters which may have different expression profiles, and may change the protein product of the gene by skipping initial exons [14]. We previously used the results from the FANTOM3 project to identify the promoter region of human and mouse *FBN1* [15] and *FBN2* (unpublished). However those data included few tissues showing expression of *FBN3* in humans. The current FANTOM5 project involves many more tissue and cell types likely to express members of the fibrillin and LTBP families [16]. We have now taken advantage of the extensive information on gene expression, promoter usage and regulation available through FANTOM5 to identify the promoter regions, possible enhancer sequences and potential regulatory motifs for each of these genes in human. We show that different promoters and enhancers are used in a tissue-specific manner under differential regulation, with predominant expression in a subset of mesenchymally derived cells but additional expression in cells of neural, embryonic and other origins, highlighting the varied roles of this gene family. This information may lead to strategies for treatment of diseases that result from mutation of these genes, such as Marfan syndrome (OMIM: 154700; *FBN1* OMIM: 134797), congenital contractural arachnodactyly (OMIM: 121050; *FBN2* OMIM: 612570), primary congenital glaucoma (OMIM: 613086; *LTBP2* OMIM: 602091) and cutis laxa with severe systemic abnormalities (OMIM: 613177; *LTBP4* OMIM: 604710) [7].

This work is part of the FANTOM5 project. Data downloads, genomic tools and co-published manuscripts have been summarized at http://fantom.gsc.riken.jp/5/.

## 2 Methods

### 2.1 Cell lines and cell culture

Five human cell lines and a primary adult derived mesenchymal stem cell culture were used to analyze expression patterns of fibrillin/LTBP gene family members. SAOS-2 (sarcoma osteogenic 2) is a hypotriploid line derived from the osteosarcoma of an 11-year-old Caucasian female and has been in culture since 1973 [17,18]. It retains many of the characteristics of osteoblasts including the ability to mineralize in the presence of phosphate molecules [18]. MG63 is a hypotriploid line derived from the osteosarcoma of a 14-year-old Caucasian male and has been in culture since 1977 [19,20]. This line fails to mineralize under experimental conditions and may represent a more immature, less osteoblastic cell line when compared to Saos-2 [21,22]. Saos-2 and MG63 cells were cultured in McCoys5A medium (Gibco) with 10% heat inactivated fetal bovine serum, antibiotics and antifungals. Normal human dermal fibroblast (NHDF) cells, grown from human circumcision material, were obtained from Dr Finn Grey, Roslin Institute. The cells were cultured in DMEM with 10% non-heat inactivated fetal bovine serum, antibiotics and antifungals. The cells were removed from the flasks with 1 × Trypsin–EDTA solution (Sigma). All cell lines were seeded at approximately 25% of confluence and processed for RNA extraction at two days after plating for the osteosarcoma cell lines and seven days after plating for NHDF.

### 2.2 Analysis of RNA and protein expression

Expression of fibrillin/LTBP family members was initially examined in cell lines using quantitative reverse transcriptase PCR from RNA extracted from the cell lines described above. In addition RNA was extracted from cell pellets from Day 2 of embryoid body formation from two human ES cell lines, H1 [23] and RH1 [24] (provided respectively by Professor Lesley Forester and Dr Paul De Sousa, Scottish Centre for Regenerative Medicine, University of Edinburgh), and Day 2 of culture of primary adipose derived mesenchymal stem cells (ADMSC) (provided by Dr. Paul DeSousa). Total RNA was extracted from cell lines using RNA-Bee (Amsbio, Abingdon, UK; http://www.amsbio.com/rna-bee.aspx). RNA was quantified using a Nanodrop spectrophotometer and cDNA was synthesized using MMLV reverse transcriptase (Promega Corporation, Madison WI, USA) and an annealing temperature of 60 °C [25]. Primers for quantitative PCR were designed using Roche primer design (Roche Diagnostics Ltd., Burgess Hill, UK; http://www.roche-applied-science.com, Supplementary Table 1). Human *GAPDH* was used as the reference gene (Qiagen Ltd., Crawley, UK; Hs_GAPDH_2_SG). Quantitative PCR was performed in triplicate using LightCycler 480 SYBR Green 1 Master Mix (Roche) on the LightCycler 480 machine (Roche). Products were quantified using software supplied by the manufacturer.

Expression of fibrillin/LTBP family members was initially examined in cell lines using quantitative reverse transcriptase PCR from RNA extracted from the cell lines described above. In addition RNA was extracted from cell pellets from Day 2 of embryoid body formation from two human ES cell lines, H1 [23] and RH1 [24] (provided respectively by Professor Lesley Forester and Dr Paul De Sousa, Scottish Centre for Regenerative Medicine, University of Edinburgh), and Day 2 of culture of primary adipose derived mesenchymal stem cells (ADMSC) (provided by Dr. Paul DeSousa). Total RNA was extracted from cell lines using RNA-Bee (Amsbio, Abingdon, UK; http://www.amsbio.com/rna-bee.aspx). RNA was quantified using a Nanodrop spectrophotometer and cDNA was synthesized using MMLV reverse transcriptase (Promega Corporation, Madison WI, USA) and an annealing temperature of 60 °C [25]. Primers for quantitative PCR were designed using Roche primer design (Roche Diagnostics Ltd., Burgess Hill, UK; http://www.roche-applied-science.com, Supplementary Table 1). Human *GAPDH* was used as the reference gene (Qiagen Ltd., Crawley, UK; Hs_GAPDH_2_SG). Quantitative PCR was performed in triplicate using LightCycler 480 SYBR Green 1 Master Mix (Roche) on the LightCycler 480 machine (Roche). Products were quantified using software supplied by the manufacturer.

The presence of the encoded proteins was ascertained using immunofluorescence with a range of antibodies. 20,000 NHDF cells per well were seeded into an eight well chamber slide (NUNC). After 7 days, the cells were fixed with 95% ethanol and 5% acetic acid for 20 min and washed with PBS. The samples were blocked with 1% BSA in PBS for 1.5 h prior to addition of a 1:100 dilution of the primary antibody and incubated over night at 4 °C. Following a wash with PBS, the secondary antibody was added at 1:1000 dilution, for 1 h at room temperature. The cells were then washed with PBS, and mounted with ProGold + DAPI (Invitrogen). Samples were viewed using Zeiss LSM 710 confocal microscope and analyzed with ZEN software at standard settings (Zeiss). Details of antibodies used are supplied in Supplementary Table 1.

The presence of the encoded proteins was ascertained using immunofluorescence with a range of antibodies. 20,000 NHDF cells per well were seeded into an eight well chamber slide (NUNC). After 7 days, the cells were fixed with 95% ethanol and 5% acetic acid for 20 min and washed with PBS. The samples were blocked with 1% BSA in PBS for 1.5 h prior to addition of a 1:100 dilution of the primary antibody and incubated over night at 4 °C. Following a wash with PBS, the secondary antibody was added at 1:1000 dilution, for 1 h at room temperature. The cells were then washed with PBS, and mounted with ProGold + DAPI (Invitrogen). Samples were viewed using Zeiss LSM 710 confocal microscope and analyzed with ZEN software at standard settings (Zeiss). Details of antibodies used are supplied in Supplementary Table 1.

### 2.3 Identification of promoter and enhancer sequences

The tissues and cells used for the FANTOM5 analysis of transcription initiation sites have been described in a parallel publication [16]. Briefly, promoters were identified using CAGE (cap analysis of gene expression) followed by Helicos high throughput sequencing [12,16,26]. Decomposition-based peak identification (DPI; [16]) was used to identify a set of transcription start sites (TSS), at permissive and robust levels (containing at least one nucleotide position with > 2 or > 10 independent single molecule observations respectively in a single library) [16]. Expression levels were determined using RLE normalized counts of CAGE tags mapping to each promoter region [16]. Promoters were then numbered by expression rank at the permissive level [16], so p1@GENE had the highest expression of all promoters associated with that gene, followed by p2@GENE etc. At the more stringent robust level some of these promoters were lost and this accounts for gaps in the numbering of robust promoters. The full permissive set can be seen on the ZENBU browser [27] (http://fantom.gsc.riken.jp/zenbu/). Composite promoters are those where two or more robust peaks occur within 100 bp of each other [16]. Identification of putative enhancers was based on the presence of bidirectional CAGE tag clusters within a 400 bp window [28]. We linked enhancers and nearby promoters (within 500 bp) with correlation tests using their expression levels across all FANTOM5 samples, requiring a Benjamini–Hochberg false discovery rate of less than 10^− 5^ [28]. We used this resource to identify the total set of robust promoters annotated to the seven fibrillin/LTPB gene family members.

### 2.4 Analysis of gene co-expression clusters

All 81 DPI-detected robust promoters for the seven genes were clustered using Biolayout *Express*^3D^ [29,30], based on CAGE-determined expression. To enter all promoters into the analysis a low cut off pair-wise Pearson correlation coefficient of 0.17 was used. Additional analysis at higher Pearson thresholds (as outlined in the Results) was performed to discover tightly coregulated promoters. The MCL inflation value was 2.2 for all analyses.

The parallel paper [16] presents clustering of 124,090 human robust promoters over all samples and in three subsets (primary cells, tissues and cell lines) based on CAGE-determined expression patterns, with Pearson correlation coefficient > 0.75 and MCL inflation value of 2.2. These data were reanalyzed for the present paper, looking for coexpression of the promoters for the seven fibrillin/LTBP genes with promoters for other genes.

### 2.5 Identification of transcription factor binding motifs and transcription factor activity

We downloaded the whole-genome alignment of the human genome with 45 other vertebrate genomes from the UCSC Genome Browser database [31]. We retained the alignments between the human, macaque, mouse, rat, cow, horse, dog, opossum, and chicken genomes, and used the T-Coffee alignment tool [32] on 1000 bp segments of the genome to optimize the alignment for the nine selected genomes. We then ran MotEvo [33] on these whole-genome alignments to predict the occurrence of transcription factor binding sites (TFBSs) in the human genome, using a background prior probability of 0.98, a prior for the UFE (unidentified functional element) [33] model of 200 relative to the weight matrices, a UFE motif length of 8 bp, and a uniform background sequence probability. Motif Activity Response Analysis (MARA) [34] was applied on these TFBS predictions to infer the regulatory network.

## 3 Results

### 3.1 Gene and protein expression of fibrilliln/LTBP family members

We used quantitative reverse transcriptase PCR (qRT-PCR) to examine expression of gene family members in cell lines including H1 and RH1 embryonic stem cells, ADMSC mesenchymal stem cells from adipose tissue, SAOS2 and MG63 osteosarcoma cell lines and NHDF fibroblasts, (Sections 2.1 and 2.2). The results are presented in Fig. 1.

*FBN1* expression was consistently high in ADMSC, NHDF and MG63 cell types but minimal in early ES cells (Fig. 1A). It was lower in SAOS2 cells. Immunocytochemistry using monoclonal antibodies for fibrillin-1 on human fibroblast cell line NHDF showed the formation of a rich extracellular fibrillar matrix (Fig. 1B). *FBN2* was highly expressed early in embryoid body formation from H1 and RH1 embryonic stem cells and in SAOS2 and NHDF cells (Fig. 1B). Immunocytochemistry using anti-fibrillin-2 antibodies showed the formation a fibrillar matrix similar to fibrillin-1 (Fig. 1D). Low but detectable expression of *FBN3* was present in both ES cell lines (H1 and RH1), with no expression in the remaining cell types (Fig. 1E), consistent with a role restricted to early development. qPCR for *LTBP1* and *LTBP4* RNA revealed some expression in cells types tested (Fig. 1F and I) while *LTBP2* was high in ADMSC and NHDF with low expression in all other cell types (Fig. 1G). *LTBP3* showed similar expression patterns except that mRNA was detectable in MG63 (Fig. 1H).

**Fig. 2:**
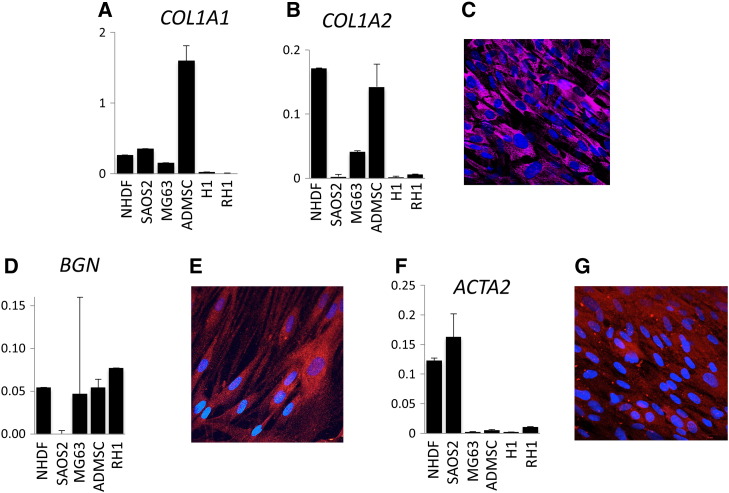
Expression of mesenchymal marker genes. qPCR was performed on RNA from cell lines SAOS2, MG63, H1 human embryonic stem cells and RH1 human embryonic stem cells and from primary cells ADMSC and NHDF. Results were normalized using *GAPDH*. See methods for more details. (A. *COL1A1*; B. *COL1A2*; D. *BGN*; F. *ACTA2*). Fluorescent immunocytochemistry was performed on Day 7 cultured human fibroblasts, NHDF. Antibody information is available in Supplementary Table 1. (C. collagen type 1; E. biglycan; G. smooth muscle actin).

These results suggested a mesenchymal distribution of most fibrillin/LTBP gene expression. To confirm this we also carried out qPCR and immunocytochemistry on the same cell lines for *COL1A1*, *COL1A2*, *BGN* and *ACTA2* (Fig. 2), genes previously shown to be highly expressed in mesenchymal cells [10]. qRT-PCR studies showed similar expression of *COL1A1* with *FBN1* and *LTBP2* while *COL1A2* and *BGN* were expressed in all mesenchymal cells examined except SAOS2 (Fig. 2). This was similar to *LTBP1* except that the latter was also highly expressed in SAOS2. The smooth muscle actin gene *ACTA2*, was highly expressed in NHDF and SAOS2 (Fig. 2D). We have previously shown high expression of this gene in a range of mesenchymal cell types [10,35]. Immunocytochemistry showed positive staining of the fibroblast cell line for collagen type1, although the protein remained intracellular (Fig. 2C), which was also seen previously for collagen 1 staining of osteosarcoma cell line MG63 [22]. Consistent with the RNA expression pattern, immunocytochemistry revealed strong staining for biglycan in NHDF cells (Fig. 2E). Positive intracellular staining of NHDF cells for smooth muscle actin was seen (Fig. 2G). These results confirm the coexpression of fibrillin/LTBP family members with mesenchymal cell markers.

### 3.2 Identification of promoters of the fibrillin/LTBP gene family

**Fig. 3:**
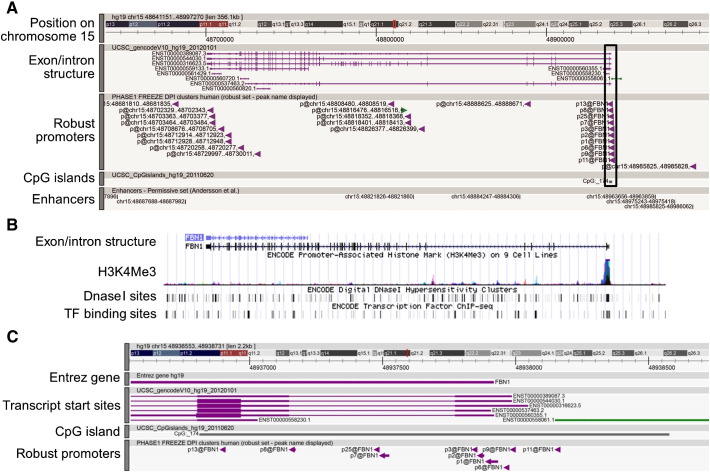
Architecture of the human *FBN1* gene. Promoters were numbered using the permissive [16] set, based on expression, with p1@FBN1 as the highest expressing promoter. Images derived from the ZENBU browser (http://fantom.gsc.riken.jp/zenbu/) and from the USCS Genome Browser (http://genome.ucsc.edu/). Images for the other gene family members are available in the Supplementary Figure or by accessing the ZENBU browser. A. The whole of the *FBN1* gene, showing the physical location, exon/intron structure (introns —horizontal lines; exons —vertical lines), robust promoters, CpG islands, and enhancers. Boxed area shows the region enlarged in C. B. Regulatory elements detected for *FBN1* by the ENCODE Project [36], showing trimethylation of lysine 4 in Histone 3 (H3K4Me3), DNase I hypersensitivity sites and transcription factor binding sites detected by chromatin immunoprecipitation and sequencing. The *FBN1* gene is aligned to the image in A. C. Enlargement of the region containing the promoters for *FBN1*, showing the consensus gene start site (Entrez gene), the start sites of known transcripts, the CpG island and the promoters in this region.

To assess further the tissue distribution and regulation of gene family members, we used FANTOM5 CAGE-based data for promoter expression. There were between 1 (*FBN3*) and 21 (*LTBP2*) robust promoters peaks associated with members of the gene family. Fig. 3 shows the location and architecture of the promoters for *FBN1* along with regulatory elements detected by the ENCODE project [36]: trimethylation of the lysine at position 4 of histone 3 (H3K4Me3), DNase I hypersensitivity sites and chromatin immunoprecipitation results for transcription factors. The same data for promoters of all family members are shown in the Supplementary Figure.

To assess further the tissue distribution and regulation of gene family members, we used FANTOM5 CAGE-based data for promoter expression. There were between 1 (*FBN3*) and 21 (*LTBP2*) robust promoters peaks associated with members of the gene family. Fig. 3 shows the location and architecture of the promoters for *FBN1* along with regulatory elements detected by the ENCODE project [36]: trimethylation of the lysine at position 4 of histone 3 (H3K4Me3), DNase I hypersensitivity sites and chromatin immunoprecipitation results for transcription factors. The same data for promoters of all family members are shown in the Supplementary Figure.

Almost all the promoters were broad promoters in CpG islands and there were no TATA box promoters (Supplementary Figure and Supplementary Table 2). The majority of robust peaks were within 100 bp of another peak, forming 18 composite promoters [16] across the gene family (Supplementary Table 2). The frequency of singleton peaks was significantly lower than the 42% seen across the whole human genome [16] (18/81; Χ^2^_1_ = 13.0; p = 0.0003) and this was attributable to *LTBP1* (0/11 singletons; Χ^2^_1_ = 7.9; p = 0.005), *LTBP2* (2/21 singletons; Χ^2^_1_ = 9.1; p = 0.0025) and *FBN2* (1/10 singletons; Χ^2^_1_ = 4.2; p = 0.04).

Almost all the promoters were broad promoters in CpG islands and there were no TATA box promoters (Supplementary Figure and Supplementary Table 2). The majority of robust peaks were within 100 bp of another peak, forming 18 composite promoters [16] across the gene family (Supplementary Table 2). The frequency of singleton peaks was significantly lower than the 42% seen across the whole human genome [16] (18/81; Χ^2^_1_ = 13.0; p = 0.0003) and this was attributable to *LTBP1* (0/11 singletons; Χ^2^_1_ = 7.9; p = 0.005), *LTBP2* (2/21 singletons; Χ^2^_1_ = 9.1; p = 0.0025) and *FBN2* (1/10 singletons; Χ^2^_1_ = 4.2; p = 0.04).

There were known transcripts (RefSeq, Ensembl ENST number etc.) starting in the region of the majority of the detected promoters, and most known alternative transcripts were supported by this analysis (Supplementary Table 2). Notably, promoters for the three alternative first exons of *FBN1* [37,38] were detected (Fig. 3). The vast majority of *FBN1* CAGE tags localized to the main composite promoter region containing p1@FBN1 (Exon A), but p11@FBN1 is probably associated with the rarely used Exon B of previous reports [37,38], while promoters p7@FBN1 and p25@FBN1 may be associated with the previously described Exon C [38] (Fig. 3B). p14@FBN2 and p18@FBN2, found in intron 25 of *FBN2* (Supplementary Figure: *FBN2* A. —red box and D.), were associated with the short transcript ENST00000507835.1. Two promoters were identified in the 3′ noncoding region of the *LTBP2* gene (p12@LTBP2 and p27@LTBP2; Supplementary Figure: *LTBP2* A. —red box). They may be associated with a short overlapping transcript from *LOC730019*.

There were known transcripts (RefSeq, Ensembl ENST number etc.) starting in the region of the majority of the detected promoters, and most known alternative transcripts were supported by this analysis (Supplementary Table 2). Notably, promoters for the three alternative first exons of *FBN1* [37,38] were detected (Fig. 3). The vast majority of *FBN1* CAGE tags localized to the main composite promoter region containing p1@FBN1 (Exon A), but p11@FBN1 is probably associated with the rarely used Exon B of previous reports [37,38], while promoters p7@FBN1 and p25@FBN1 may be associated with the previously described Exon C [38] (Fig. 3B). p14@FBN2 and p18@FBN2, found in intron 25 of *FBN2* (Supplementary Figure: *FBN2* A. —red box and D.), were associated with the short transcript ENST00000507835.1. Two promoters were identified in the 3′ noncoding region of the *LTBP2* gene (p12@LTBP2 and p27@LTBP2; Supplementary Figure: *LTBP2* A. —red box). They may be associated with a short overlapping transcript from *LOC730019*.

Some published transcripts were not supported by CAGE tags. For example, there was no support at the robust level for a promoter for the long *FBN2* transcript ENST00000508053.1 which has an additional six 5′ non-coding exons (data not shown; available at http://fantom.gsc.riken.jp/zenbu/), nor were there promoters for two long transcripts (AY203940 and AK022050AY203940AK022050) which overlap *FBN3*. Similarly there was no evidence for a promoter for the longest reported *LTBP4* transcript, NM_003573 (Supplementary Figure: *LTBP4* A. —ENST00000545697 and ENST00000204005).

Some published transcripts were not supported by CAGE tags. For example, there was no support at the robust level for a promoter for the long *FBN2* transcript ENST00000508053.1 which has an additional six 5′ non-coding exons (data not shown; available at http://fantom.gsc.riken.jp/zenbu/), nor were there promoters for two long transcripts (AY203940 and AK022050AY203940AK022050) which overlap *FBN3*. Similarly there was no evidence for a promoter for the longest reported *LTBP4* transcript, NM_003573 (Supplementary Figure: *LTBP4* A. —ENST00000545697 and ENST00000204005).

Transcription start sites were also detected where there was no identified transcript. For instance, at the 5′ end of *LTBP1*, alternate transcripts with a long or short 5′ UTR have been reported (Supplementary Figure: *LTBP1* D.). However, there was no CAGE support for the transcript with the shorter UTR (ENST00000354476.3). Instead, a small TSS cluster was detected midway between the main (long) start site about 200 bp upstream of the annotated beginning of the putative shorter transcript, which may indicate that the shorter transcript is incomplete. Similarly a number of identified TSS in *LTBP3* did not correlate with known transcripts (for example, see Supplementary Figure: *LTBP3* D. and E.).

Transcription start sites were also detected where there was no identified transcript. For instance, at the 5′ end of *LTBP1*, alternate transcripts with a long or short 5′ UTR have been reported (Supplementary Figure: *LTBP1* D.). However, there was no CAGE support for the transcript with the shorter UTR (ENST00000354476.3). Instead, a small TSS cluster was detected midway between the main (long) start site about 200 bp upstream of the annotated beginning of the putative shorter transcript, which may indicate that the shorter transcript is incomplete. Similarly a number of identified TSS in *LTBP3* did not correlate with known transcripts (for example, see Supplementary Figure: *LTBP3* D. and E.).

Although most promoters were located in CpG islands, there was little conservation of sequence around the promoters. Many of the promoter regions themselves were pyrimidine-rich (on the coding strand). The coding strand of the main *FBN1* promoter region contained a characteristic pyrimidine (T-rich) stretch, about 70 nucleotides upstream of the transcription start site associated with p1@FBN1 [15,37,39]. The main *FBN2* promoter was in a C-rich region but the promoter region of the alternative transcript (p14@FBN2 and p18@FBN2) was not in a CpG island and was AT rich. The *FBN3* promoter was also pyrimidine rich but shared no sequence homology with the other genes. The region around p1@LTBP1 contained alternating strings of purines and of pyrimidines.

The major promoters were all supported by regulatory elements detected in the ENCODE project [36] (Fig. 3, Supplementary Figure: panel B for each gene). Where regulatory elements were not detected this may result from lack of expression in the cell types used in the ENCODE project. For example, there are no ENCODE elements for the composite promoter containing p14@FBN2 and p18@FBN2 (Supplementary Figure: *FBN2* A. —red box compared with B.), which is almost exclusively expressed in testis, a tissue type not represented in the cell lines examined in the ENCODE Project.

The major promoters were all supported by regulatory elements detected in the ENCODE project [36] (Fig. 3, Supplementary Figure: panel B for each gene). Where regulatory elements were not detected this may result from lack of expression in the cell types used in the ENCODE project. For example, there are no ENCODE elements for the composite promoter containing p14@FBN2 and p18@FBN2 (Supplementary Figure: *FBN2* A. —red box compared with B.), which is almost exclusively expressed in testis, a tissue type not represented in the cell lines examined in the ENCODE Project.

### 3.3 Enhancers correlated with promoters of the fibrillin/LTBP gene family

Expression correlation tests, using pairs of expression peaks within 500 kb of each other (Section 2.3), predicted between four and 10 enhancers for the genes, with one to seven predicted enhancers per promoter (Supplementary Table 3). Putative enhancers were located as close as 2 kb up- or downstream of the promoter region. There was no significantly associated possible enhancer activity for the most 5′ promoters of *LTBP1* (p3@LTBP1, p7@LTBP1, p8@LTBP1, p9@LTBP1, p10@LTBP1 and p15@LTBP1). Promoters within a compound promoter were associated with the same predicted enhancer(s) (Table 1, Supplementary Tables 2 and 3). All promoters for *FBN1*, *LTBP2* and the single *FBN3* promoter had enhancers which could be putatively linked with the promoter by means of expression correlations (see Section 2.3), while about half of the promoters for the other genes lacked any significant association with enhancer expression.

Expression correlation tests, using pairs of expression peaks within 500 kb of each other (Section 2.3), predicted between four and 10 enhancers for the genes, with one to seven predicted enhancers per promoter (Supplementary Table 3). Putative enhancers were located as close as 2 kb up- or downstream of the promoter region. There was no significantly associated possible enhancer activity for the most 5′ promoters of *LTBP1* (p3@LTBP1, p7@LTBP1, p8@LTBP1, p9@LTBP1, p10@LTBP1 and p15@LTBP1). Promoters within a compound promoter were associated with the same predicted enhancer(s) (Table 1, Supplementary Tables 2 and 3). All promoters for *FBN1*, *LTBP2* and the single *FBN3* promoter had enhancers which could be putatively linked with the promoter by means of expression correlations (see Section 2.3), while about half of the promoters for the other genes lacked any significant association with enhancer expression.

p7@FBN1 and p25@FBN1, initiating the alternative first exon, Exon C [38], were associated with different putative enhancers from p1@FBN1 and associated promoters. p14@FBN2 and p18@FBN2, in intron 25 of *FBN2*, were correlated with a putative enhancer expression peak about 460 kb downstream of the promoter (Supplementary Table 3). This enhancer was not correlated with the other *FBN2* promoters. p12@LTBP2 was uniquely associated with a bidirectional expression peak 50 kb upstream (Supplementary Table 3). In contrast, p10@LTBP3, p12@LTBP3 and p21@LTBP3 located within the second and third exons of the major *LTBP3* transcript were correlated with one of the same predicted enhancers as the major promoter region containing p1@LTBP3. For *LTBP4*, each promoter tended to be associated with a different enhancer.

p7@FBN1 and p25@FBN1, initiating the alternative first exon, Exon C [38], were associated with different putative enhancers from p1@FBN1 and associated promoters. p14@FBN2 and p18@FBN2, in intron 25 of *FBN2*, were correlated with a putative enhancer expression peak about 460 kb downstream of the promoter (Supplementary Table 3). This enhancer was not correlated with the other *FBN2* promoters. p12@LTBP2 was uniquely associated with a bidirectional expression peak 50 kb upstream (Supplementary Table 3). In contrast, p10@LTBP3, p12@LTBP3 and p21@LTBP3 located within the second and third exons of the major *LTBP3* transcript were correlated with one of the same predicted enhancers as the major promoter region containing p1@LTBP3. For *LTBP4*, each promoter tended to be associated with a different enhancer.

### 3.4 Tissue specificity of promoters of fibrillin/LTBP family members

The promoters all showed the characteristics of tissue restricted expression, as defined in the parallel paper [16]. Specifically, 76% of FBN promoters and 50% of LTBP promoters had a median expression level across the 889 cell and tissue types of 0 and for all promoters the maximum expression was substantially greater than 10 times the median (Supplementary Table 2).

The promoters all showed the characteristics of tissue restricted expression, as defined in the parallel paper [16]. Specifically, 76% of FBN promoters and 50% of LTBP promoters had a median expression level across the 889 cell and tissue types of 0 and for all promoters the maximum expression was substantially greater than 10 times the median (Supplementary Table 2).

To investigate this tissue restricted expression further, we used a number of ontologies [16] to classify the tissues and cell types with maximum expression of the promoters (FANTOM5 Resource Browser, http://fantom.gsc.riken.jp/5/sstar/ and Supplementary Table 4). Promoters in one composite cluster within a gene tended to be expressed in tissues associated with the same ontology terms (homogeneous composite promoters [16]), and promoters in separate regions of the same gene were often associated with different ontology terms, indicating promoter switches associated with cell-type specificity.

To investigate this tissue restricted expression further, we used a number of ontologies [16] to classify the tissues and cell types with maximum expression of the promoters (FANTOM5 SSTAR http://fantom.gsc.riken.jp/5/sstar/ and Supplementary Table 4). Promoters in one composite cluster within a gene tended to be expressed in tissues associated with the same ontology terms (homogeneous composite promoters [16]), and promoters in separate regions of the same gene were often associated with different ontology terms, indicating promoter switches associated with cell-type specificity.

In general, the major promoters of all gene family members other than *FBN3* were strongly expressed in cells associated with ontology terms indicating mesenchymal origin (Table 1, Supplementary Table 4). The strongest promoters of *FBN1*, p1@FBN1 and p2@FBN1, were highly expressed in cells of the same ontology terms (including *fibroblast*, *mesodermal cell*, *dense mesenchyme tissue* and a number of muscle-related terms) (Supplementary Table 4). Similar ontology terms were found for the main promoters of *FBN2*, which was expressed in a range of cells from fetal and embryonic tissues, fibroblasts, osteoblasts, placenta, hair follicle and lens epithelial cells (Table 1). p2@LTBP4 shared many terms with *FBN1* and *FBN2* (for example, *fibroblast*, *muscle cell*, *myoblast*) (Supplementary Table 4). The main composite promoter region of *LTBP1* (containing p1@LTBP1; Supplementary Figure: *LTBP1* C.) was highly expressed in adult heart samples and also expressed in tissue and cells types of mesenchymal origin. This composite promoter was associated with mesenchyme ontology terms including *fibroblast* but also with many muscle related terms (Supplementary Table 4). Although the promoter regions identified in *LTBP2* were associated with some generic mesenchymal ontology terms (such as *fibroblast* and *myoblast*), the high expression levels in cardiovascular cell types also led to a preponderance of terms associated with the vasculature (*vascular associated smooth muscle cell*, *vascular system*, *blood vessel*, *vascular cord*) (Supplementary Table 4).

In general, the major promoters of all gene family members other than *FBN3* were strongly expressed in cells associated with ontology terms indicating mesenchymal origin (Table 1, Supplementary Table 4). The strongest promoters of *FBN1*, p1@FBN1 and p2@FBN1, were highly expressed in cells of the same ontology terms (including *fibroblast*, *mesodermal cell*, *dense mesenchyme tissue* and a number of muscle-related terms) (Supplementary Table 4). Similar ontology terms were found for the main promoters of *FBN2*, which was expressed in a range of cells from fetal and embryonic tissues, fibroblasts, osteoblasts, placenta, hair follicle and lens epithelial cells (Table 1). p2@LTBP4 shared many terms with *FBN1* and *FBN2* (for example, *fibroblast*, *muscle cell*, *myoblast*) (Supplementary Table 4). The main composite promoter region of *LTBP1* (containing p1@LTBP1; Supplementary Figure: *LTBP1* C.) was highly expressed in adult heart samples and also expressed in tissue and cells types of mesenchymal origin. This composite promoter was associated with mesenchyme ontology terms including *fibroblast* but also with many muscle related terms (Supplementary Table 4). Although the promoter regions identified for *LTBP2* were associated with some generic mesenchymal ontology terms (such as *fibroblast* and *myoblast*), the high expression levels in cardiovascular cell types also led to a preponderance of terms associated with the vasculature (*vascular associated smooth muscle cell*, *vascular system*, *blood vessel*, *vascular cord*) (Supplementary Table 4).

A number of promoters were expressed in tissues of other origins. Thus p7@FBN1 and p1@LTBP3 were enriched in ontology terms reflecting development of the nervous system (*presumptive neural plate*, *neural tube*, *neural rod*, *structure with developmental contribution from neural crest*, *future spinal cord*) (Supplementary Table 4). p3@FBN2 peaked in hematopoietic cell types, and was associated with ontology terms such as *classical monocyte* as well as terms reflecting mesenchyme/mesoderm expression (Supplementary Table 4). p2@LTBP2 was also expressed in some hematopoietic cells while p3@LTBP4 was associated with lymphocytes of the immune system (ontology terms *mature alpha*-*beta T cell*, *immature T cell*) but also with terms associated with other organs (*craniocervical region*, *skin of body*, *surface structure*). p7@LTBP2 demonstrated significant expression levels in both hematopoietic and epithelial samples and was enriched for terms reflection this expression pattern (*endoepithelial cell*, *epithelial cell*) (Supplementary Table 4). The high expression promoter of *LTBP4*, p1@LTBP4, was enriched in ontology terms reflecting an endodermal origin. p14 @FBN2 and p18@FBN2, located in intron 25, (Supplementary Figure) displayed highest expression in testis and both promoters were associated with ontology terms such as *testis*, *male reproductive organ*, *gonad* and *external genitalia*/*reproductive organ* (Supplementary Table 4).

A number of promoters were expressed in tissues of other origins. Thus p7@FBN1 and p1@LTBP3 were enriched in ontology terms reflecting development of the nervous system (*presumptive neural plate*, *neural tube*, *neural rod*, *structure with developmental contribution from neural crest*, *future spinal cord*) (Supplementary Table 4). p3@FBN2 peaked in hematopoietic cell types, and was associated with ontology terms such as *classical monocyte* as well as terms reflecting mesenchyme/mesoderm expression (Supplementary Table 4). p2@LTBP2 was also expressed in some hematopoietic cells while p3@LTBP4 was associated with lymphocytes of the immune system (ontology terms *mature alpha*-*beta T cell*, *immature T cell*) but also with terms associated with other organs (*craniocervical region*, *skin of body*, *surface structure*). p7@LTBP2 demonstrated significant expression levels in both hematopoietic and epithelial samples and was enriched for terms reflecting this expression pattern (*endoepithelial cell*, *epithelial cell*) (Supplementary Table 4). The high expression promoter of *LTBP4*, p1@LTBP4, was enriched in ontology terms reflecting an endodermal origin. p14 @FBN2 and p18@FBN2, located in intron 25, (Supplementary Figure) displayed highest expression in testis and both promoters were associated with ontology terms such as *testis*, *male reproductive organ*, *gonad* and *external genitalia*/*reproductive organ* (Supplementary Table 4).

Some promoters were associated with early development. The sole robust promoter for *FBN3*, p2@FBN3, was almost exclusively expressed in cells of fetal and embryonic origin. This expression pattern was associated with ontology terms such as *embryonic stem cell*, *neuronal stem cell*, *neuroectodermal cell* as well as some terms relating to pigmentation (*melanocyte*, *melanoblast* and *pigment cell*) (Supplementary Table 4l). Promoters of *LTBP1* leading to the longer transcript (Supplementary Figure) were highly expressed in embryonic and extraembryonic cell/tissue types including chorionic and amniotic membrane cells, fetal heart, epithelial and endothelial tissue samples. Within this region, p3@LTBP1 had the highest activity, and transcription from this region was found primarily in fetal heart. It was most strongly associated with ontology terms such as *extraembryonic cell*/*structure*, *mesenchyme*, *compound organ* and *membranous layer* (Supplementary Table 4).

Some promoters were associated with early development. The sole robust promoter for *FBN3*, p2@FBN3, was almost exclusively expressed in cells of fetal and embryonic origin. This expression pattern was associated with ontology terms such as *embryonic stem cell*, *neuronal stem cell*, *neuroectodermal cell* as well as some terms relating to pigmentation (*melanocyte*, *melanoblast* and *pigment cell*) (Supplementary Table 4l). Promoters of *LTBP1* leading to the longer transcript (Supplementary Figure) were highly expressed in embryonic and extraembryonic cell/tissue types including chorionic and amniotic membrane cells, fetal heart, epithelial and endothelial tissue samples. Within this region, p3@LTBP1 had the highest activity, and transcription from this region was found primarily in fetal heart. It was most strongly associated with ontology terms such as *extraembryonic cell*/*structure*, *mesenchyme*, *compound organ* and *membranous layer* (Supplementary Table 4).

Variability of promoter use in different cell types was supported by the H3K4Me3 track from the ENCODE data (Supplementary Figure), where different colored peaks (representing trimethylation levels in different cell types) can be seen for different promoters. For example, the composite *LTBP1* promoter containing p1@LTBP1 showed strong trimethylation of H3K4 in human lung fibroblasts (NHLF), while the composite promoter containing p3@LTBP1 was strongly trimethylated in most of the cell lines, except embryonic stem cells and a human lymphoblastoid cell line (Gm12878) (Supplementary Figure: *LTBP1* B.).

Variability of promoter use in different cell types was supported by the H3K4Me3 track from the ENCODE data (Supplementary Figure), where different colored peaks (representing trimethylation levels in different cell types) can be seen for different promoters. For example, the composite *LTBP1* promoter containing p1@LTBP1 showed strong trimethylation of H3K4 in human lung fibroblasts (NHLF), while the composite promoter containing p3@LTBP1 was strongly trimethylated in most of the cell lines, except embryonic stem cells and a human lymphoblastoid cell line (Gm12878) (Supplementary Figure: *LTBP1* B.).

### 3.5 Coexpression of fibrillin/LTBP family members

**Fig. 4:**
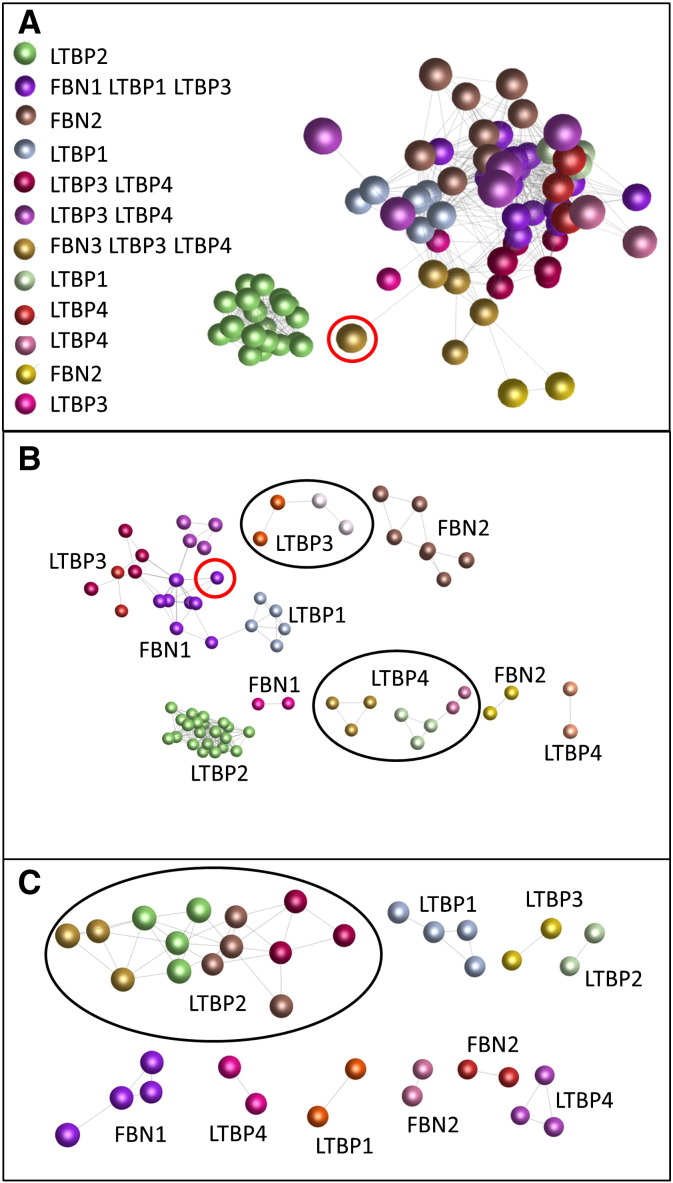
Coexpression of promoters of the fibrillin/LTBP gene family. Colored spheres (nodes) represent different promoters and the lines between them show correlated expression patterns at the threshold indicated. Spheres of the same color form a coexpression cluster of promoters with expression more similar to each other than to the other promoters in the analysis. Promoters within each of the clusters are listed in Supplementary Table 2. A. Clustering of expression pattern at low correlation coefficient threshold (0.17) using Biolayout *Express*^3D^. All 81 promoters were included. Genes whose promoters are within the different colored clusters are shown in the legend. p2@FBN3 (circled in red) clustered with p11@LTBP3, p1@LTBP4, p9@LTBP4 and p13@LTBP4. B. Clustering at moderate correlation coefficient threshold (0.50). Seventy promoters were included. Genes whose promoters are within the different colored clusters are shown on the figure. Promoters for most genes formed separate clusters, except that p6@LTBP1 (circled in red) grouped with *FBN1* promoters. Two *LTBP3* clusters and three *LTBP4* clusters formed separate closely related groups (circled in black), although there were also other distinct clusters containing promoters for these genes. C. Clustering at high correlation coefficient threshold (0.75). Twenty-five promoters were included. Genes whose promoters are within the different colored clusters are shown on the figure. Promoters for all genes formed separate clusters with four distinct but closely related clusters (circled in black) for *LTBP2*.

To explore further the similarities and differences in promoter expression across the gene family, we used RLE-normalized tag counts [16] to analyze the level of expression of promoters for all seven gene family members across the 889 RNA samples from human tissues, primary cells and cell lines previously described [16] (data available in Supplementary Table 2). CAGE-determined expression levels for each of the 81 promoters were entered into Biolayout *Express*^3D^. Biolayout *Express*^3D^ employs a statistical approach to look at transcript-to-transcript similarities in expression pattern across the samples analyzed, by calculation of a Pearson pairwise correlation matrix [29,30]. To enter all promoters into the analysis, a very low Pearson correlation coefficient threshold of 0.17 was used initially. Fig. 4A shows each promoter as a node (sphere) and the edges (lines) between them represent pairwise Pearson correlation coefficients of ≥ 0.17. All *LTBP2* promoters formed a separate cluster (Cluster 1) with no edges to promoters for any of the other genes, even at this low correlation level. This indicates that the *LTBP2* promoters are more similar in expression pattern to each other than to any other promoters. *FBN2* promoters formed two separate clusters (Clusters 3 and 11) and *LTBP1* promoters were also in two clusters (Clusters 4 and 8) suggesting two patterns of expression for these genes. In contrast, at this low level of correlation *LTBP3* and *LTBP4* promoters were frequently in the same cluster (Clusters 5 and 7), or associated with the single *FBN3* promoter (Cluster 6), showing that these promoters share some similarity of expression pattern. Some *LTBP3* and *LTBP4* promoters formed separate distinct clusters (Cluster 12 and Clusters 9 and 10 respectively). One *LTBP1* and six *LTBP3* promoters also clustered with the *FBN1* promoters (Cluster 2).

To explore further the similarities and differences in promoter expression across the gene family, we used RLE-normalized tag counts [16] to analyze the level of expression of promoters for all seven gene family members across the 889 RNA samples from human tissues, primary cells and cell lines previously described [16] (data available in Supplementary Table 2). CAGE-determined expression levels for each of the 81 promoters were entered into Biolayout *Express*^3D^. Biolayout *Express*^3D^ employs a statistical approach to look at transcript-to-transcript similarities in expression pattern across the samples analyzed, by calculation of a Pearson pairwise correlation matrix [29,30]. To enter all promoters into the analysis, a very low Pearson correlation coefficient threshold of 0.17 was used initially. Fig. 4A shows each promoter as a node (sphere) and the edges (lines) between them represent pairwise Pearson correlation coefficients of ≥ 0.17. All *LTBP2* promoters formed a separate cluster (Cluster 1) with no edges to promoters for any of the other genes, even at this low correlation level. This indicates that the *LTBP2* promoters are more similar in expression pattern to each other than to any other promoters. *FBN2* promoters formed two separate clusters (Clusters 3 and 11) and *LTBP1* promoters were also in two clusters (Clusters 4 and 8) suggesting two patterns of expression for these genes. In contrast, at this low level of correlation *LTBP3* and *LTBP4* promoters were frequently in the same cluster (Clusters 5 and 7), or associated with the single *FBN3* promoter (Cluster 6), showing that these promoters share some similarity of expression pattern. Some *LTBP3* and *LTBP4* promoters formed separate distinct clusters (Cluster 12 and Clusters 9 and 10 respectively). One *LTBP1* and six *LTBP3* promoters also clustered with the *FBN1* promoters (Cluster 2).

At a Pearson correlation coefficient threshold of 0.5, 11 promoters were excluded from the analysis indicating that their expression pattern was sufficiently different from any other promoter that they did not correlate at this level. The sole *FBN3* promoter, p2@FBN3, was in this excluded group as were a promoter for *FBN2* and *LTBP1*, seven for *LTBP3* and two for *LTBP4*. Nine of these 11 were singleton promoters while two were within a composite promoter. The network of associations at P ≥ 0.5 is shown in Fig. 4B. All *LTBP2* promoters continued to cluster together (Cluster 1) while *FBN1* (Clusters 2 and 12), *FBN2* (Clusters 3 and 11) and *LTBP1* (Clusters 4 and 6) formed two clusters each. p6@LTBP1 clustered with the majority of the *FBN1* promoters, indicating an expression pattern more similar to *FBN1* than to the other *LTBP1* promoters. p7@FBN1 and p25@FBN1, initiating the alternative first exon, Exon C [38] clustered separately from the other *FBN1* promoters. *LTBP3* (Clusters 5, 9, 13 and 15) and *LTBP4* (Clusters 7, 8, 10 and 14) formed four clusters each. These results suggest that the most diverse expression patterns were found for promoters of *LTBP3* and *LTBP4*, while *LTBP2* had the most similar expression pattern across all promoters.

The grouping of the promoters was also assessed at a Pearson correlation threshold of 0.75. At this level, only 25 promoters were included in the analysis, indicating that the expression of the majority did not correlate with any other at a correlation coefficient of ≥ 0.75. The remaining tight clusters consisted of four clusters of *LTBP2* promoters and single clusters for *FBN1*, *LTPB1* and *LTBP4* (Fig. 4C).

### 3.6 Identification of genes co-expressed with promoters for fibrillin/LTBP family members

The results in Sections 3.1, 3.4 and 3.5 established that the fibrillin/LTBP gene family members tend to be expressed in cells of mesenchymal origin, although some promoters were associated with expression in diverse other cell types. To obtain further insight into the variability of expression patterns, we analyzed coexpression with the full set of promoters determined by CAGE, performed for the previous study [16], which presented a network of 4664 coexpression groups derived from expression profiles of 182,000 promoters across 887 primary cell types, tissues and cancer cell lines (Fig. 7 in [16]). The analysis was performed both for the entire sample and for the subsets of tissues, cell lines and primary cells. This enabled us to identify promoters that were co-expressed with members of the fibrillin/LTBP gene family (data shown in Supplementary Table 2).

The results in Sections 3.1, 3.4 and 3.5 established that the fibrillin/LTBP gene family members tend to be expressed in cells of mesenchymal origin, although some promoters were associated with expression in diverse other cell types. To obtain further insight into the variability of expression patterns, we analyzed coexpression with the full set of promoters determined by CAGE, performed for the previous study [16], which presented a network of 4664 coexpression groups derived from expression profiles of 182,000 promoters across 887 primary cell types, tissues and cancer cell lines (Fig. 7 in [16]). The analysis was performed both for the entire sample and for the subsets of tissues, cell lines and primary cells. This enabled us to identify promoters that were co-expressed with members of the fibrillin/LTBP gene family (data shown in Supplementary Table 2).

Overall, promoters for the gene family members tended to cluster with promoters for genes such as *ACTA2*, *BGN*, *CALU*, *CCDC80*, *COL1A1*, *COL1A2*, *COL3A1 COL4A1*, *COL5A1*, *COL6A1*, *COL7A1*, *COL17A1*, *COL21A1*, *DCN*, *ELN*, *FBLN2*, *FBLN5*, *FN1*, *LOXL3*, *PCOLCE*, *POSTN*, *SOST*, *SPARC*, *TGFB1*, *TGFBR3* and *THBS1* which have previously been identified to be expressed in mesenchymal cell types [10]. However, many promoters failed to cluster, or clustered only with other promoters for the same gene, indicating distinctive expression patterns that were not shared by other genes.

p1@FBN1, p2@FBN1, p3@FBN1 and p9@FBN1 tended to cluster together with a small number of other promoters such as promoters for *CALU* and *CCDC80*. In contrast, the promoters associated with the alternative exon C, p7@FBN1 and p25@FBN1 clustered with promoters for neurally expressed genes. Similarly the promoters for the short testis specific *FBN2* transcript, p14@FBN2 and p18@FBN2, clustered together with testis specific and connective tissue genes; the remaining *FBN2* promoters frequently failed to cluster. p2@FBN3 was found in large clusters of promoters for genes involved in regulating development including *NANOG*, *FOXG1*, *FOXO6*, *FOXP1*, *PRICKLE1* and *SOX14* as well as neural genes such as potassium channels and *MAPT* and connective tissue genes including *COL9A1*, *COL9A3*, *ACTA1* and *ACTN3*.

Promoters for LTBP genes tended to fail to cluster or form clusters with other promoters for the same gene, particularly in the primary cell analysis. p1@LTBP1, p2@LTBP1, p4@LTBP1 and p5@LTBP1 formed a small cluster when all samples were analyzed and p3@LTBP1, p7@LTBP1 and p8@LTBP1 formed a cluster when primary cells were analyzed (Supplementary Table 2). However some LTBP promoters clustered with promoters for mesenchymal genes and occasionally were found with promoters for other members of the fibrillin–LTBP gene family. *LTBP3* promoters usually failed to cluster, even with each other. The coexpression analysis for *LTBP4* promoters consistently grouped p2@LTBP4, p6@LTBP4 and p7@LTBP4 in clusters containing only these promoters (Supplementary Table 2l).

Promoters for LTBP genes tended to fail to cluster or formed clusters with other promoters for the same gene, particularly in the primary cell analysis. p1@LTBP1, p2@LTBP1, p4@LTBP1 and p5@LTBP1 formed a small cluster when all samples were analyzed and p3@LTBP1, p7@LTBP1 and p8@LTBP1 formed a cluster when primary cells were analyzed (Supplementary Table 2). However some LTBP promoters clustered with promoters for mesenchymal genes and occasionally were found with promoters for other members of the fibrillin/LTBP gene family. *LTBP3* promoters usually failed to cluster, even with each other. The coexpression analysis for *LTBP4* promoters consistently grouped p2@LTBP4, p6@LTBP4 and p7@LTBP4 in clusters containing only these promoters (Supplementary Table 2).

### 3.7 Transcription factors regulating fibrillin/LTBP gene family members

We used Motif Activity Response Analysis (MARA) [34] to infer the regulatory network of transcription factor motifs in 47 promoters of the fibrillin/LTBP gene family (Supplementary Table 2). Motifs associated with the MAZ, SP1, GTF2I and KLF4 transcription factors were found to regulate 16 to 20 promoters. For *FBN1* (seven promoters in total), the motifs for GTF2I, NKX3-1, TBX4-5 and TFAP4 regulated five promoters, while for *FBN2* (6 promoters) HIF1A was found to regulate four promoters and TFDP1, KLF4, TFAP2B and TOPORS regulated three promoters. There were no significant regulatory edges for *FBN3* (Supplementary Table 2l). However, binding motifs for the transcription factors MAZ, PATZ1, RREB1, SP1 and TFAP4 were detected in the promoter region of *FBN3*.

We used Motif Activity Response Analysis (MARA) [34] to infer the regulatory network of transcription factor motifs in 47 promoters of the fibrillin/LTBP gene family (Supplementary Table 2). Motifs associated with the MAZ, SP1, GTF2I and KLF4 transcription factors were found to regulate 16 to 20 promoters. For *FBN1* (seven regulated promoters in total), the motifs for GTF2I, NKX3-1, TBX4-5 and TFAP4 regulated five promoters, while for *FBN2* (6 promoters) HIF1A was found to regulate four promoters and TFDP1, KLF4, TFAP2B and TOPORS regulated three promoters. There were no significant regulatory edges for *FBN3* (Supplementary Table 2). However, binding motifs for the transcription factors MAZ, PATZ1, RREB1, SP1 and TFAP4 were detected in the promoter region of *FBN3*.

*LTBP1* (regulated at nine promoters in total) and *LTBP2* (regulated at 16 promoters) showed significant regulation by the MAZ motif in seven and eight promoters respectively. Five promoters of *LTBP1* were also regulated by HIC1 and NFY while four promoters were regulated by PRDM1, RXR and SOX 17. LTBP2 was predicted to be regulated by KLF4 (at 10 promoters), SP1 (at eight promoters), GATA4 and TEAD (at five promoters) and XCPE1 (at four promoters) was associated with *LTBP2*. Regulation at seven *LTBP3* promoters was detected, through motifs for GTF2I and SP1 (at six promoters), MED-1 (at five promoters) and TFAP2 and ZFP161 (at four promoters). Only two *LTBP4* promoters were significantly associated with motifs, with TRAF2B found for both (Supplementary Table 2).

*LTBP1* (regulated at nine promoters in total) and *LTBP2* (regulated at 16 promoters) showed significant regulation by the MAZ motif in seven and eight promoters respectively. Five promoters of *LTBP1* were also regulated by HIC1 and NFY while four promoters were regulated by PRDM1, RXR and SOX 17. LTBP2 was predicted to be regulated by KLF4 (at 10 promoters), SP1 (at eight promoters), GATA4 and TEAD (at five promoters) and XCPE1 (at four promoters) was associated with *LTBP2*. Regulation at seven *LTBP3* promoters was detected, through motifs for GTF2I and SP1 (at six promoters), MED-1 (at five promoters) and TFAP2 and ZFP161 (at four promoters). Only two *LTBP4* promoters were significantly associated with motifs, with TRAF2B found for both (Supplementary Table 2).

## 4 Discussion

Members of the fibrillin/LTBP gene family appear to play two roles in the extracellular matrix. Firstly, they contribute structurally to the formation of the matrix. In particular, fibrillin-1 and fibrillin-2 are integral components of the 10 nm microfibrils which provide strength and elasticity to the matrix [40,41]. Secondly, they are instrumental in regulating the bioavailability of members of the TGFβ superfamily of growth factors [6]. While some fibrillin/LTBP family members (fibrillin-1, fibrillin-2 and LTBP2) do not appear to bind TGFβ molecules directly, they interact with other members of the family that do bind to these growth factors. The TGFβ superfamily contains 33 proteins [42,43] and it is possible that fibrillin/LTBP family members bind directly to other TGFβ-like molecules such as bone morphogenic proteins (BMPs) and activins/inhibins. TGFβ activation requires the action of force on the TGFβ prodomain, and this may be exerted via fibrillins reacting to perturbations of the ECM [44]. Therefore these proteins are an important component of the extracellular matrix both in maintaining the structural integrity of the matrix and in controlling the growth and differentiation of the cells they surround. Their overlapping roles raise the possibility that they can substitute for each other in some circumstances, providing some resilience to inactivating mutations in family members.

In this study we have examined gene expression and regulation of the seven fibrillin/LTBP family members using results from the FANTOM5 CAGE analysis, a genome wide analysis of transcription start sites and bidirectional putative enhancers [16,28]. We have demonstrated the predominant expression of this gene family in mesenchyme-derived cells and tissues. It is also clear that some gene family members are required in early development, in neural tissue and the cardiovascular system. Expression derived from CAGE tag analysis was confirmed for a number of mesenchymal and embryonic stem cell lines using quantitative reverse transcriptase PCR and immunocytochemistry.

There were multiple examples of differential use of promoters in different tissue types (promoter switching) and this was frequently associated with differences in ENDOCE regulatory elements and distinctive predicted enhancer activity. The main promoter region of *FBN1* was highly expressed in a range of connective tissue cells, consistent with previously published transcription factor activity [10,15,37] but p7@FBN1 expression was predominantly found in neural cell types enriched in ontology terms related to neural development, clustered with other neural-associated genes and was associated with a unique enhancer. This promoter showed regulation by FOXD3 (Supplementary Table 2) a transcriptional regulator essential for progenitor cells and found mainly within the embryonic neural crest [45].

There were multiple examples of differential use of promoters in different tissue types (promoter switching) and this was frequently associated with differences in ENDOCE regulatory elements and distinctive predicted enhancer activity. The main promoter region of *FBN1* was highly expressed in a range of connective tissue cells, consistent with previously published transcription factor activity [10,15,37] but p7@FBN1 expression was predominantly found in neural cell types enriched in ontology terms related to neural development, clustered with other neural-associated genes and was associated with a unique enhancer. This promoter showed regulation by FOXD3 (Supplementary Table 2) a transcriptional regulator essential for progenitor cells and found mainly within the embryonic neural crest [45].

Promoter switching with a change in tissue specificity was also seen for *FBN2*, where p14@FBN2 and p18@FBN2 were expressed in reproductive cell types. The transcript associated with these promoters, ENST00000507835.1, covers coding exons 26 to 34 of the full length *FBN2* and the polypeptide would consist of eight cbEGF domains with short N- and C-terminal flanking sequences (http://www.ensembl.org). Preliminary examination of the mouse CAGE data from FANTOM5 showed an equivalent promoter exclusively expressed in mouse testis, suggesting that this transcript has a real function, perhaps involved with sequestration of calcium. Further investigation of this novel transcript should reveal more about its role.

*LTBP1* is documented to have at least two alternative transcription start regions (http//:www.ensembl.org) and this switch in promoters was confirmed in our analysis. One promoter region demonstrated high expression in early development and low expression in mesenchymal cells (p10@LTBP1, p8@LTBP1, p9@LTBP1, p3@LTBP1, p15@LTBP1 and p7@LTBP1) while the alternate promoter region (p6@LTBP1, p2@LTBP1, p1@LTBP1,p5@LTBP1 and p4@LTBP1) was associated with mesenchyme-related ontology terms and expressed in heart. The major promoters p1@LTBP1 and p2@LTBP1 were associated with the same transcription factor motifs (LHX, GTF2I, PRDM1, SOX17), while the alternative promoter region was associated with a distinct set of transcription factors motifs (including HIC1, NFY, NR1H4).

Putative enhancers within 500 kb of the promoter regions were significantly associated with many of the promoters examined. The majority were downstream of the promoter region and some were as close 2 kb from the promoter. In general many promoters in one gene were correlated with the same predicted enhancers although there were a few examples of promoter-specific enhancers, associated with promoters of unique expression pattern.

Cluster analysis of expression from the different promoters highlighted the differences between the gene family members. Members of a composite promoter often clustered together, but few promoters for different genes were found in the same cluster. For example, most promoters for *FBN2* showed similar expression patterns and ontology term enrichment to *FBN1*. However, *FBN1* and *FBN2* promoters did not cluster together in the co-expression analyses and there was little overlap of transcription factor motif activity between *FBN1* and *FBN2* promoters, suggesting that there were subtle differences in their expression patterns and regulation. In particular, *FBN2* promoters tended to be associated with the epithelial and pluripotency transcription factor KLF4 [46] and other stem cell associated factors TFAP2B [47] and PAX5 [48,49] consistent with a requirement for fibrillin-2 in early development. In contrast, *FBN1* promoters were associated with mesenchymal transcription factors [10].

Although there was no transcription factor that was associated with all family members, there was apparent regulation across the group through a number of transcription factor motifs including those for MAZ, SP1, GTF2I and KLF4. Regulation through transcription factor motifs reflecting tissue specificity was also found. The group of promoters between p1@LTBP2 and p15@LTBP2 (Supplementary Figure: *LTBP2* A —blue box and C.), for example, showed significant potential to bind GATA4 (associated with cardiac differentiation [50,51]) and MYOD (myogenic differentiation; reviewed in [52]). FANTOM5 data for these promoters showed highest expression in aortic smooth muscle, and various other mesenchyme specific tissue and cell types.

Although there was no transcription factor that was associated with all family members, there was apparent regulation across the group through a number of transcription factor motifs including those for MAZ, SP1, GTF2I and KLF4. Regulation through transcription factor motifs reflecting tissue specificity was also found. The group of promoters between p1@LTBP2 and p15@LTBP2 (Supplementary Figure: *LTBP2* A —blue box and C.), for example, showed significant potential to bind GATA4 (associated with cardiac differentiation [50,51]) and MYOD (myogenic differentiation; reviewed in [52]). FANTOM5 data for these promoters showed highest expression in aortic smooth muscle cells, and various other mesenchyme specific tissue and cell types.

Because its expression is restricted to fetal and embryonic tissue types [9], the promoter of *FBN3* could not be determined from the earlier FANTOM3 data which lacked samples from relevant human cells. FANTOM5 has a much greater representation of human cells and tissues from early developmental stages and we have now identified the promoter region and the most common starting nucleotide for human *FBN3*. We have confirmed that fibrililn-3 is expressed in early development and neural tissues of humans [9]. Since *Fbn3* is inactive in mouse and rats [9], it is possible that one of the other gene family members substitutes for its function. Since transcription factor binding motifs found in the promoter region of *FBN3* overlap with those of LTBP genes but not fibrillin genes, it is conceivable that its role is taken by one of the LTBPs. From our analysis possible candidates based on expression pattern in human embryonic cell types would be *Ltbp1* (through p3@LTBP1) or *Ltbp4* (through p5@LTBP4). Preliminary analysis of FANTOM5 data for mouse indicates that the equivalent of p3@LTBP1 is strongly expressed in embryonic tissue, especially neuron derived neurospheres, while a region equivalent to p5@LTBP4 is expressed in embryonic stem cells (data not shown; available at FANTOM5 website http://fantom.gsc.riken.jp/5/), supporting this possibility.

Mutations in human *FBN1*, *FBN2*, *LTBP2*, *LTBP3* and *LTBP4* have been associated with connective tissue disease in humans. Transgenic and natural mutation mouse models, available for *Fbn1*, *Fbn2*, *Ltbp1*, *Ltbp2*, *Ltbp3* and *Ltbp4* also show phenotypic abnormalities of connective tissue (reviewed in [7]), consistent with the predominant expression in mesenchymal cells of these genes. Further analysis of gene family members for genetic variation which affects the different promoter regions may reveal a role in more subtle connective tissue phenotypes. In addition, examination of promoter usage in relevant tissues in the presence of these mutations may reveal whether the severity of the phenotype is modified by changes in expression of gene family members, indicating a degree of redundancy. Because of similarities in structure and expression patterns, upregulation of fibrillin/LTBP family members may provide a possible therapeutic approach for these connective tissue conditions.

## 5 Conclusions

This study supports the differential roles of members of the fibrillin/LTBP gene family. We have shown the strong mesenchymal expression of most family members and most promoters and highlighted promoter and enhancer switching associated with changes in tissue specificity. We were unable to find evidence for a single “master mesenchyme regulator” but there was considerable overlap in the transcription factor activity associated with these genes. These data aid in better understanding the overall function and activity of the members of the fibrillin/LTBP family.

The following are the supplementary data related to this article

Supplementary Figure Promoters of the fibrillin/LTBP family members.

Supplementary Table 1 qPCR primers and antibodies.

Supplementary Table 2 Promoter characteristics, expression levels, transcription factor associations and expression clusters.

Supplementary Table 3 Enhancers of fibrillin/LTBP family members.

Supplementary Table 4 Ontology terms associated with cell types expressing fibrillin/LTBP family member promoters.

Supplementary data to this article can be found online at http://dx.doi.org/10.1016/j.ymgme.2013.12.006.

## Data access

The data in the paper will be freely available.

The FANTOM5 atlas is accessible from http://fantom.gsc.riken.jp/5/. FANTOM5 enhancers can also be accessed from. http://enhancer.binf.ku.dk.

## Authors' contributions

MRD carried out bioinformatic and molecular genetic analysis, participated in the design of the study and helped to draft the manuscript. RA provided the enhancer analysis. JS and NB supported the analysis and visualization. JKB performed the initial clustering of all CAGE detected promoters. MdH carried out the MARA analysis. HK managed the data handling. ARRF was responsible for FANTOM5 management and concept. KMS conceived this study, participated in its design and coordination, carried out much of the analysis and interpretation, performed the clustering analysis of the promoters and drafted the manuscript. All authors read and approved the final manuscript.

## Figures and Tables

**Table 1:** Expression levels for the highest expressing promoters of fibrillin/LTBP family members.

Promoter name	RLE normalized expression value	Cell type
p1@FBN1	2562	Smooth muscle cells (aorta)
	2496	Fibroblast (aortic adventitia)
	1437	Fibroblast (skin)
	1407	Sertoli cells
	1358	Preadipocyte (breast)
p1@FBN2	1547	Cell line: HEPM (derived from normal human embryonic palatal mesenchyme)
	1002	Fibroblast (skin)
	994	Cell line: HTST (derived from a human sacrococcigeal teratoma)
	768	Hair follicle outer root sheath cells
	753	Cell line: H-EMC-SS (derived from a human myxoid chondrosarcoma)
p2@FBN3	6	Occipital lobe (fetal)
	5	Cell line: SK-N-MC (derived from a human neuroepithelioma)
	4	H9 embryonic stem cells
	3	H9 embryonic stem cells
	3	H9 embryoid body cells
p1@LTBP1	1903	Chondrocyte, redifferentiated
	1365	Chondrocyte, redifferentiated
	1278	Mesenchymal precursor cell from bone marrow
	1107	Fibroblast (aortic adventitia)
	1051	Smooth muscle cells (aorta)
p1@LTBP2	190	Aorta (adult pooled)
	186	Dura mater (adult)
	152	Preadipocyte (breast)
	143	Chondrocyte, redifferentiated
	136	Cell line: Hs 132 (derived from a human spindle cell sarcoma)
p1@LTBP3	391	Fibroblast (aortic adventitial)
	332	Mesenchymal stem cells (adipose)
	295	Smooth muscle cells (aorta)
	246	Cardiac myocyte
	244	Aorta (adult pooled)
p1@LTBP4	391	Aorta (adult pooled)
	382	Mitral valve (adult heart)
	380	Cervix, adult
	260	Prostate (adult)
	250	Tricuspid valve (adult heart)

Expression values are RLE normalized tags per million [16].
