# Supplementary material for: Transcriptional profiling of the human fibrillin/LTBP gene family, key regulators of mesenchymal cell functions
Source: Mol Genet Metab. 2014 May;112(1):73–83. doi: 10.1016/j.ymgme.2013.12.006 (PMC4019825; doi:10.1016/j.ymgme.2013.12.006)

Davis et al

Transcriptional profiling of the human fibrillin/LTBP gene family, key regulators of mesenchymal cell functions.

Supplementary Figure

Promoter architecture for promoters of the fibrillin/LTBP gene family

Each page shows the promoter region of a member of the fibrillin/LTBP gene family.

For each gene:

Panel A shows an overview of the whole gene structure, CpG islands and enhancers in the vicinity of the gene. Genes shown in green are transcribed from forward DNA strand so the 5'end is at the left; genes shown in purple are transcribed from the reverse DNA strand, so the 5' end is at the right.

Panel B shows data from the ENCODE Project for the whole gene region, aligned with the gene image in Panel A. ENCODE data were accessed at the UCSC Genome Browser (http://genome.ucsc.edu) on 7 October 2013. Reference: The ENCODE Project Consortium, A user's guide to the Encyclopedia of DNA Elements (ENCODE) PLoS Biol 9 (2011) e1001046.

Panel C and subsequent panels show enlargement of the regions within the gene of robust promoters, including any CpG islands.

FBN1

A. Overview of the human *FBN1* region showing known transcripts, promoters, CpG islands and close enhancers. Blue boxed area shows the main promoter region (enlarged in C.)

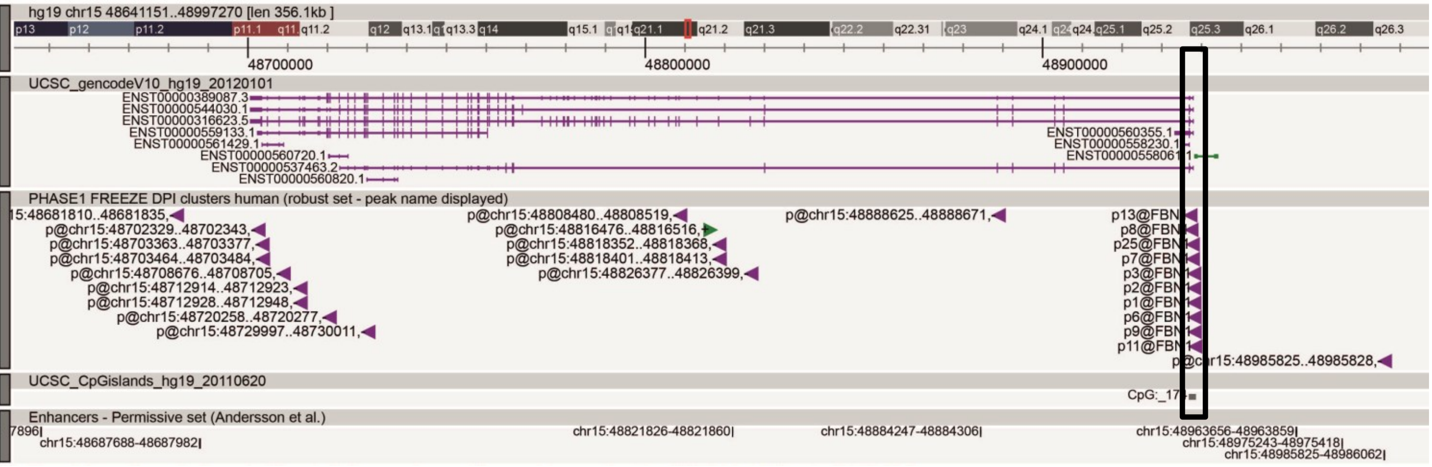

B. ENCODE regulatory elements

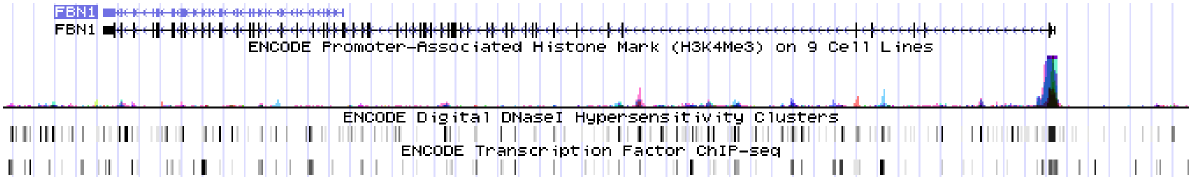

C. Enlargement of the *FBN1* promoter region

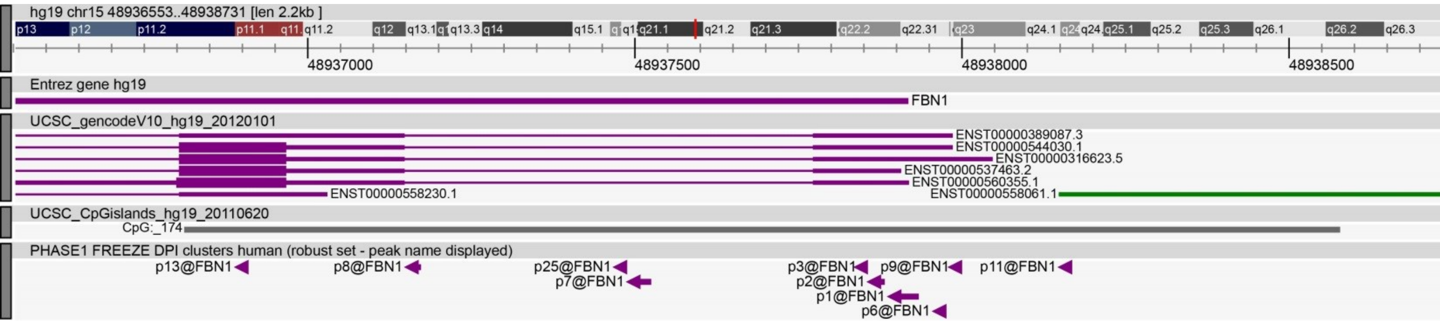

FBN2

A. Overview of the human *FBN2* region showing known transcripts, promoters, CpG islands and close enhancers. Blue boxed area shows the main promoter region (enlarged in B.) Red boxed area shows the testis specific promoter region (enlarged in C.)

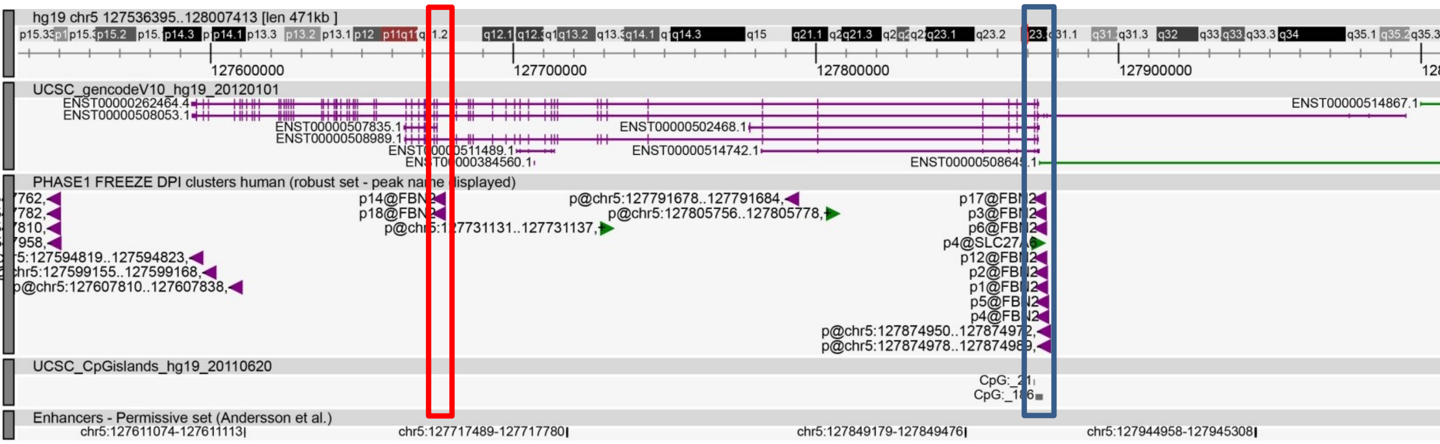

B. ENCODE regulatory elements

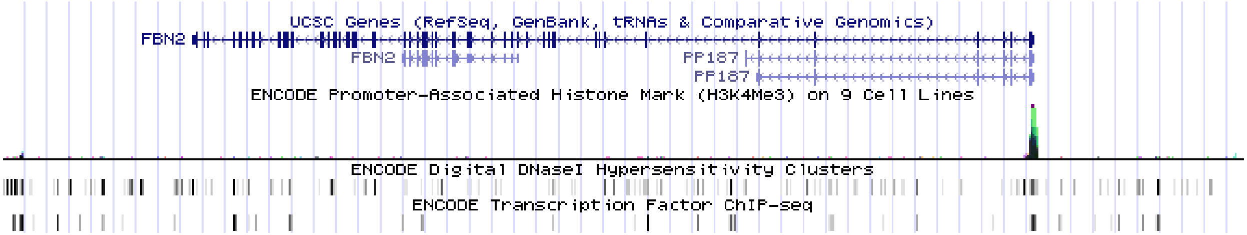

C. Enlargement of the *FBN2* promoter region

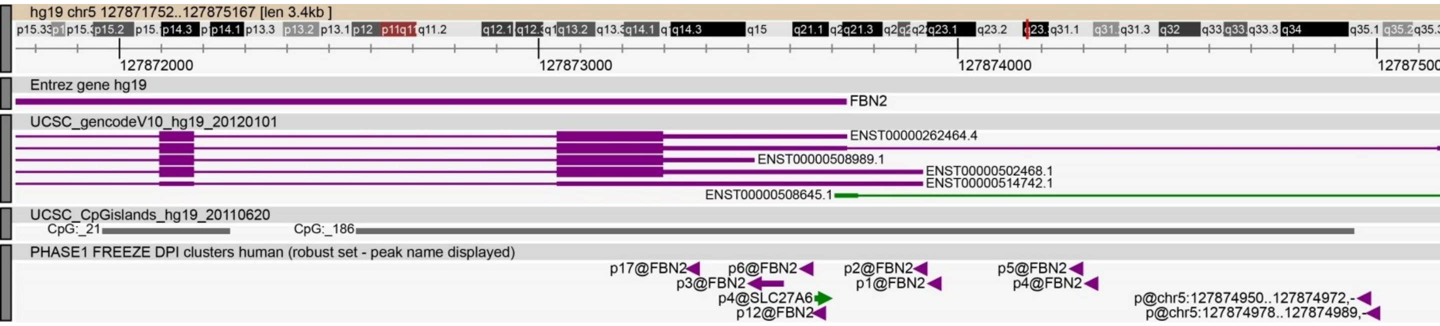

D. Enlargement of the testis specific promoter region within the *FBN2* gene

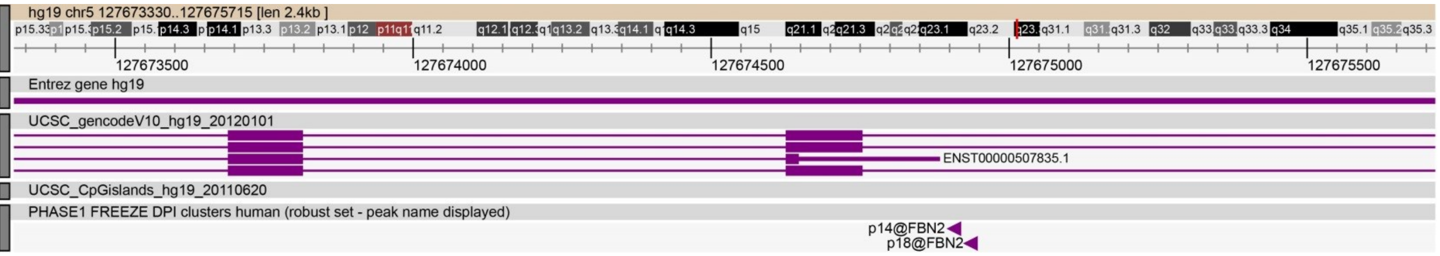

FBN3

A. Overview of the human *FBN3* region showing known transcripts, promoters, CpG islands and close enhancers. Boxed area shows the promoter region (enlarged in B.)

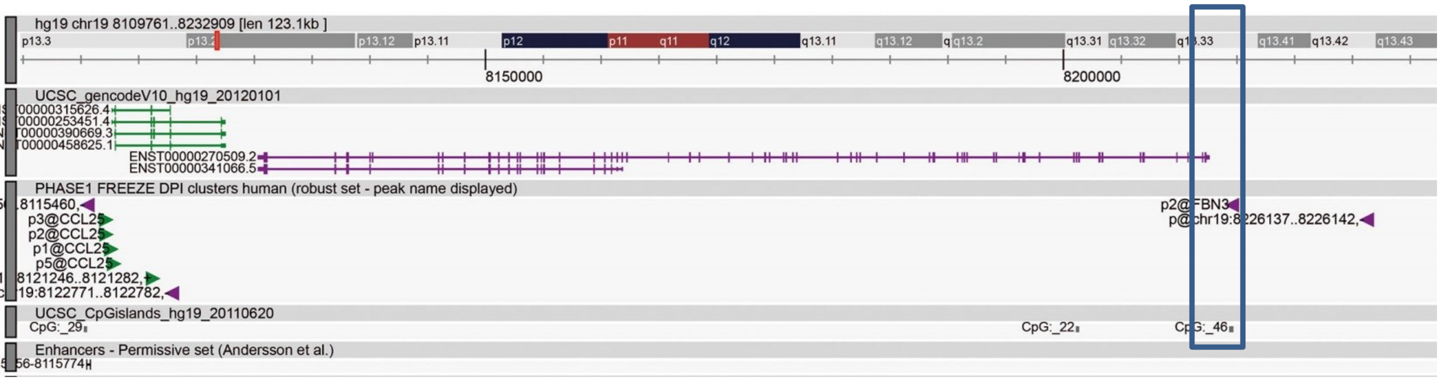

B. ENCODE regulatory elements

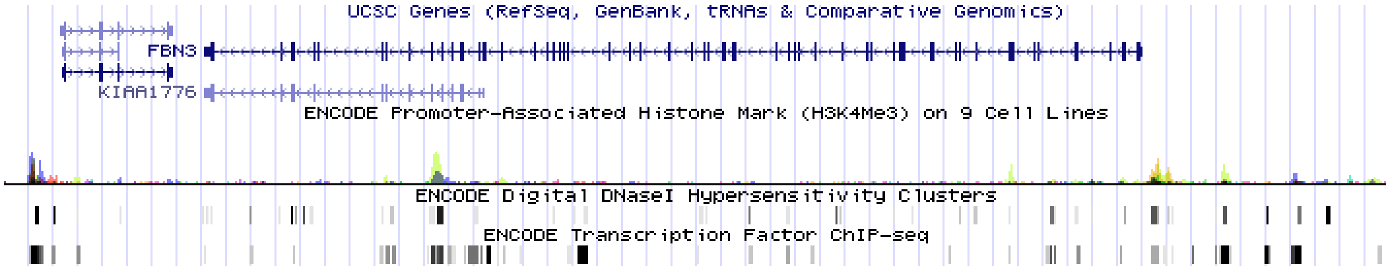

C. Enlargement of the *FBN3* promoter region

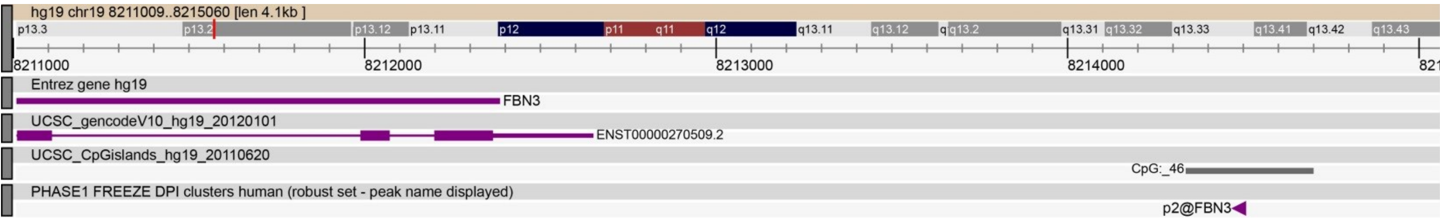

LTBP1

A. Overview of the human *LTBP1* region showing known transcripts, promoters, CpG islands and close enhancers. Blue boxed area shows the strongest promoter region (enlarged in C.) Red boxed area shows the second promoter region (enlarged in D.)

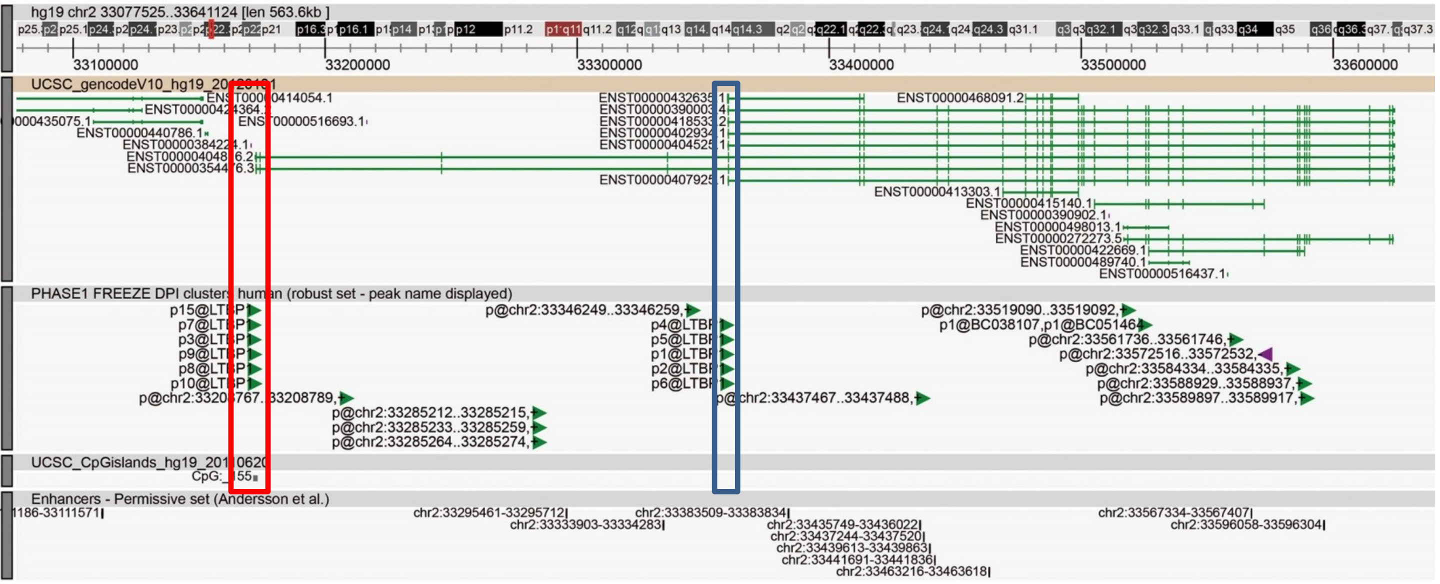

B. ENCODE regulatory elements

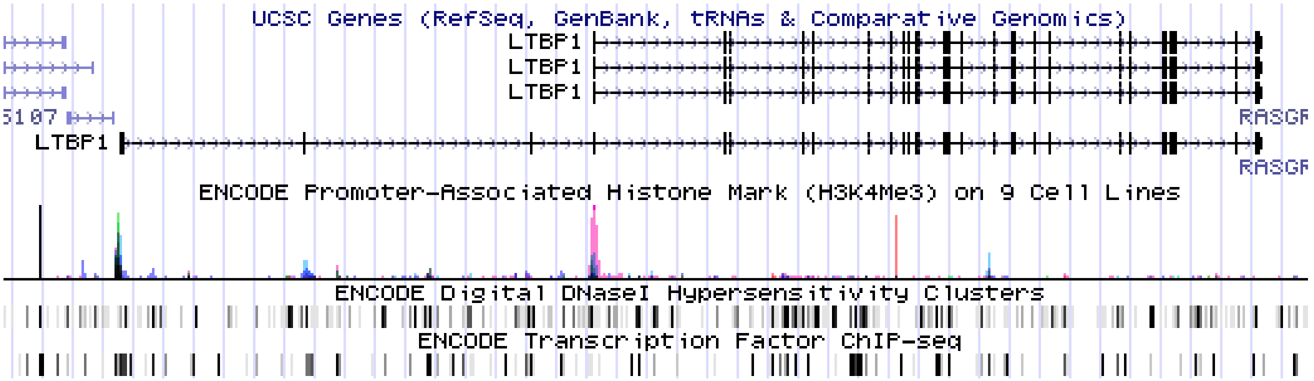

C. Enlargement of the strongest *LTBP1* promoter region

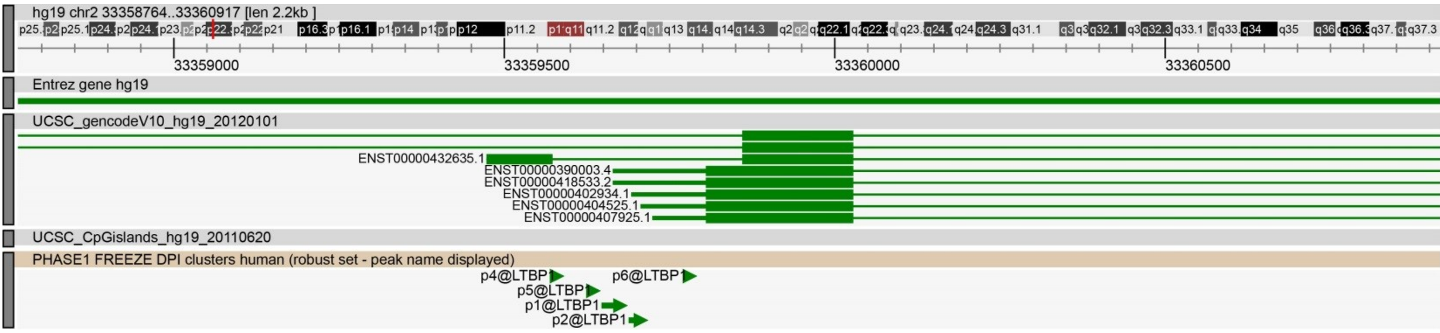

D. Enlargement of the secondary *LTBP1* promoter region

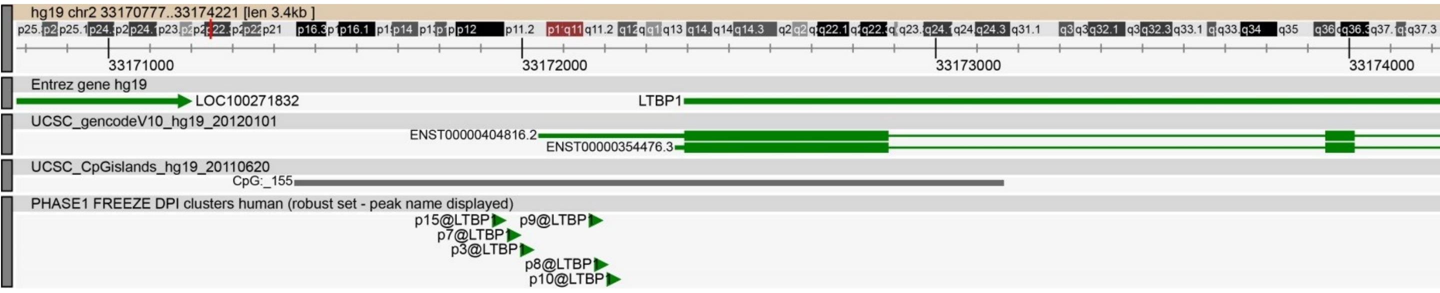

LTBP2

A. Overview of the human *LTBP2* region showing known transcripts, promoters, CpG islands and close enhancers. Blue boxed area shows the strongest promoter region (enlarged in C.) Red boxed area shows the second promoter region (enlarged in D.)

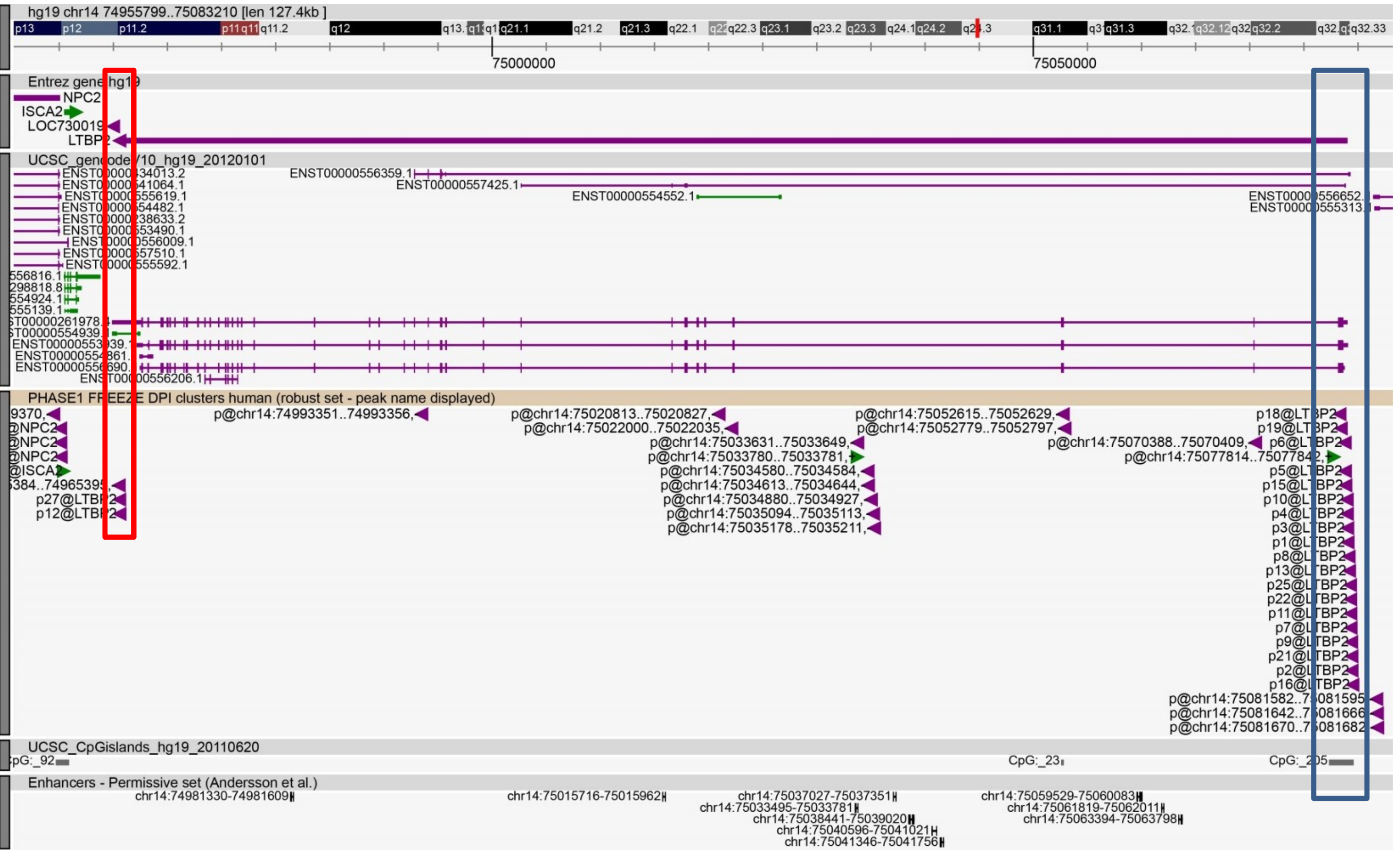

B. ENCODE regulatory elements

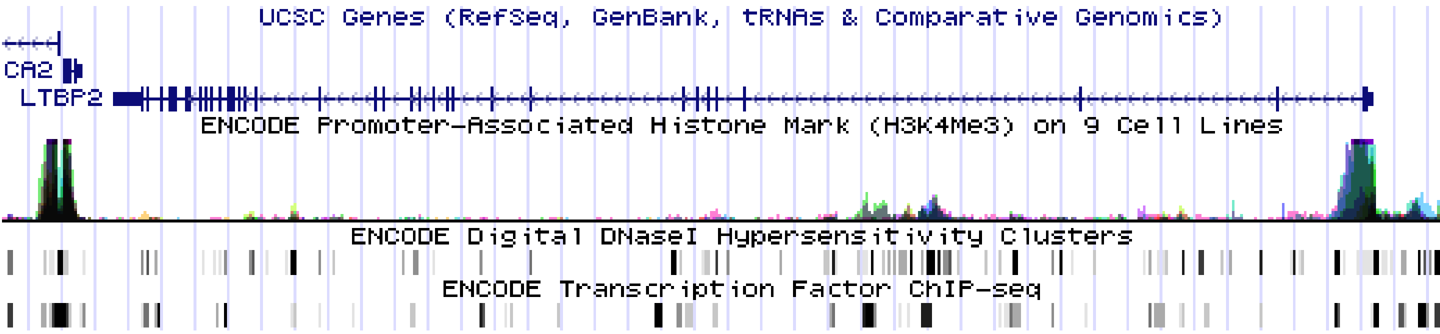

LTBP2 (continued)

C. Enlargement of the strongest *LTBP2* promoter region

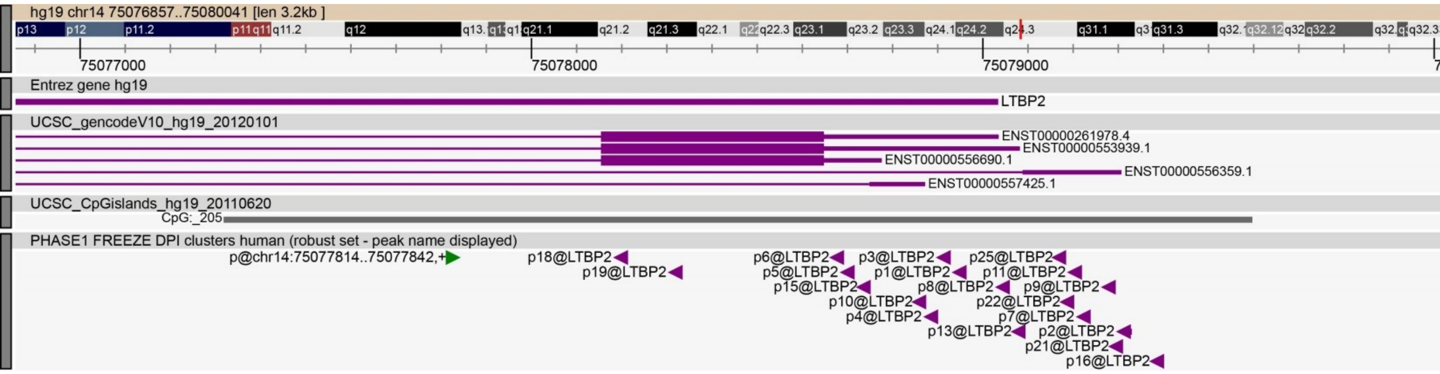

D. Enlargement of the secondary *LTBP2* promoter region

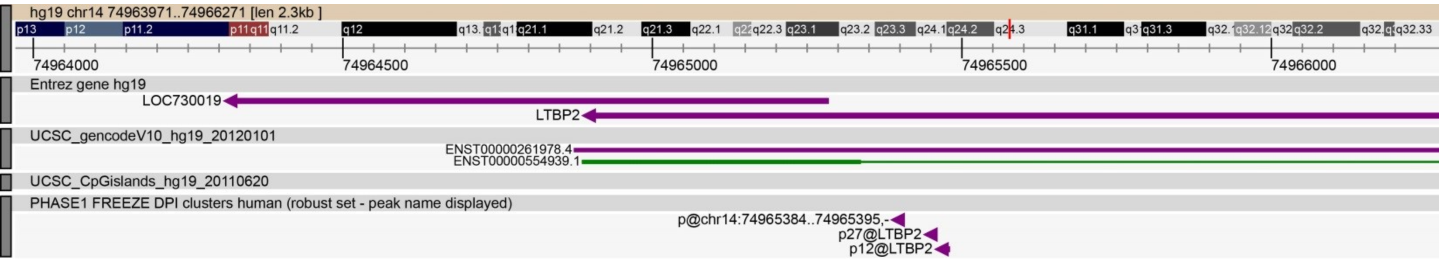

LTBP3

A. Overview of the human *LTBP3* region showing known transcripts, promoters, CpG islands and close enhancers. Blue boxed area shows the strongest promoter region (enlarged in C.) Other boxed areas show other identified promoters (red enlarged in D.; green enlarged in E.; orange enlarged in F.)

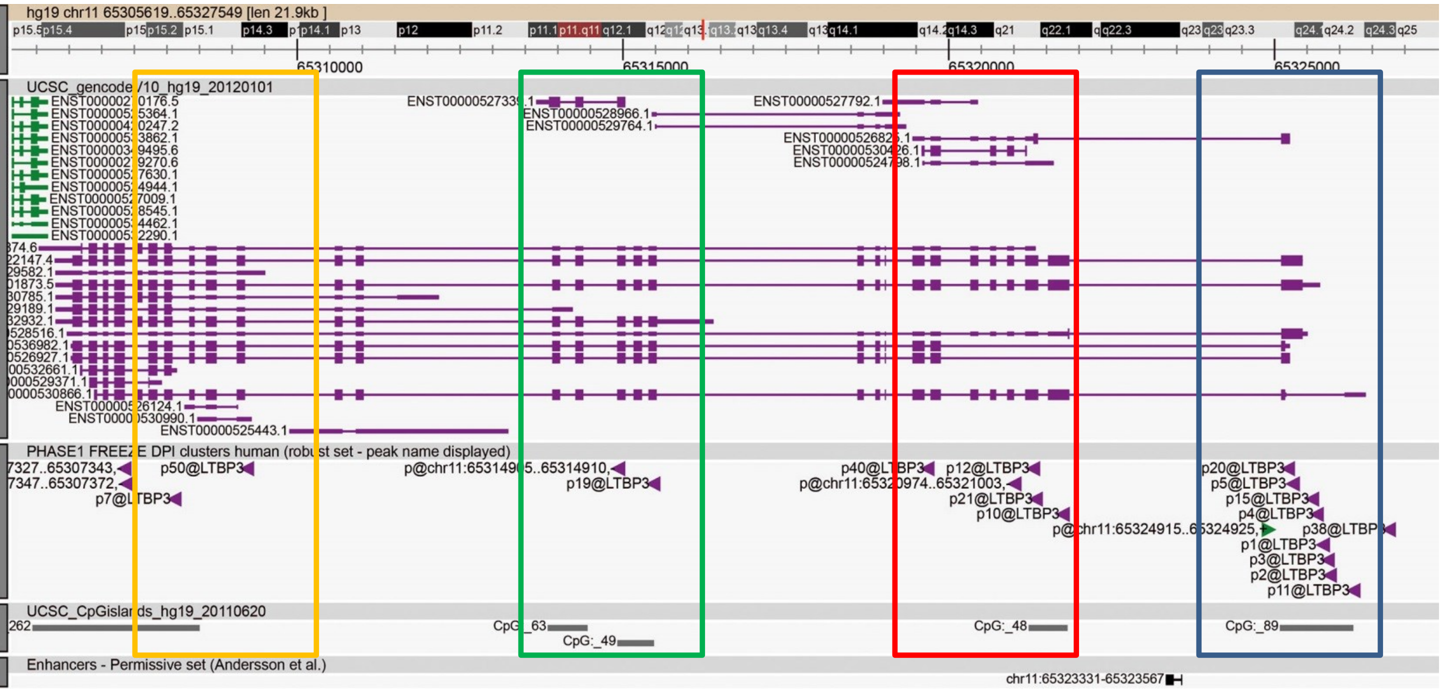

B. ENCODE regulatory elements

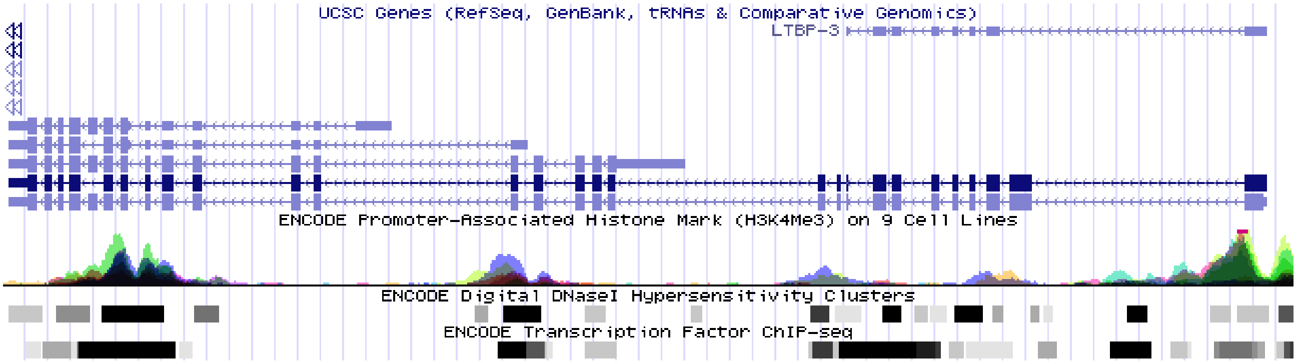

LTBP3 (continued)

C. Enlargement of the major *LTBP3* promoter region

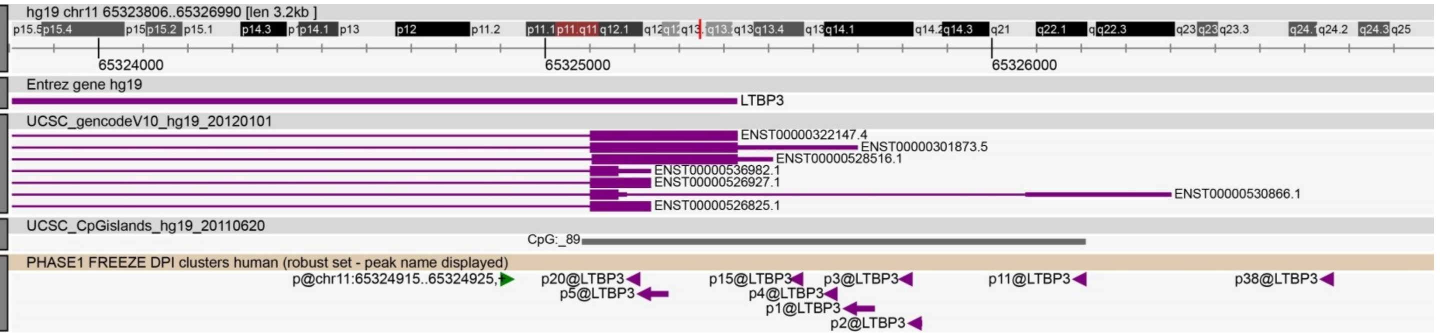

D. Enlargement of secondary *LTBP3* promoter region

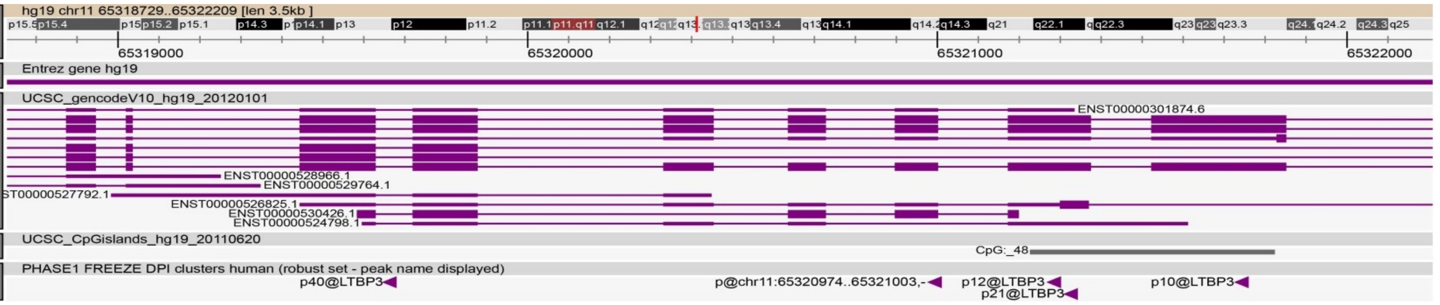

E. Enlargement of secondary *LTBP3* promoter region

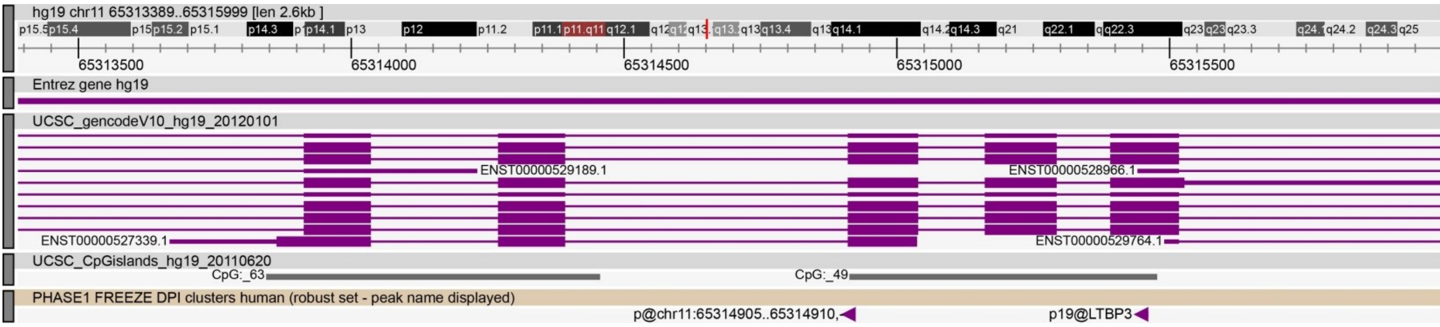

F. Enlargement of secondary *LTBP3* promoter region

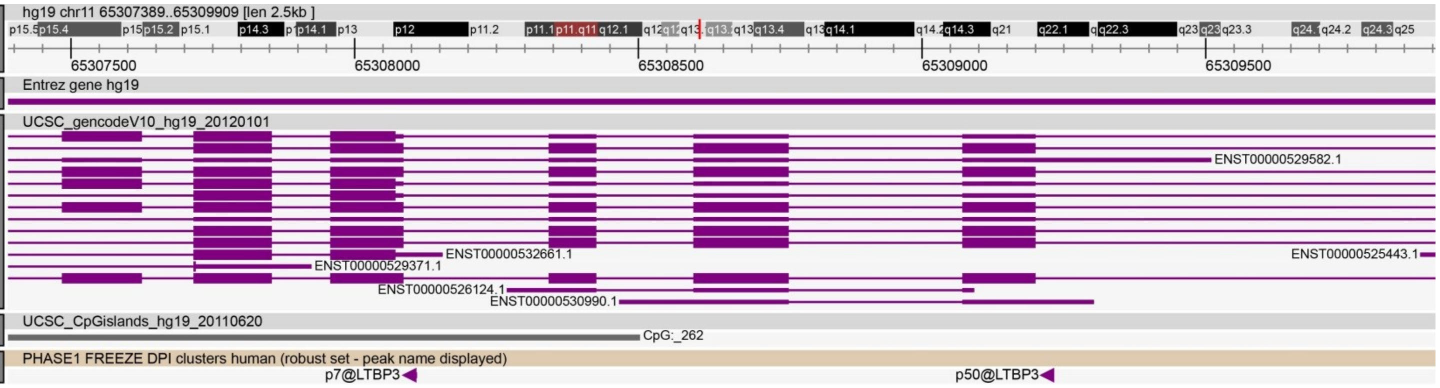

LTBP4

A. Overview of the human *LTBP4* region showing known transcripts, promoters, CpG islands and close enhancers. Blue box show the region around the strongest promoter, p1@LTBP4 which is a singleton and does not form part of a composite promoter (enlarged in B.). Other boxed areas show regions of composite promoters (red enlarged in C.; orange enlarged in D.)

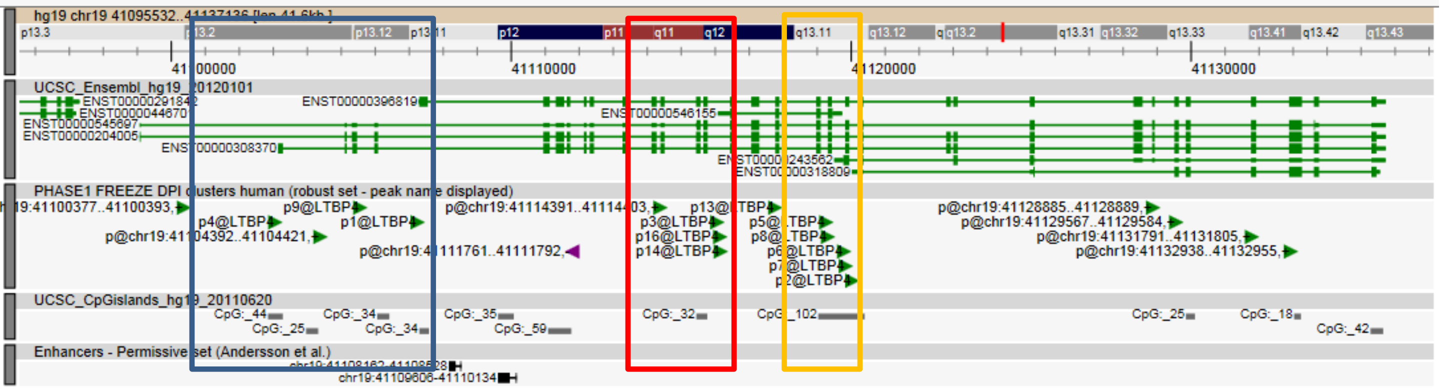

B. ENCODE regulatory elements

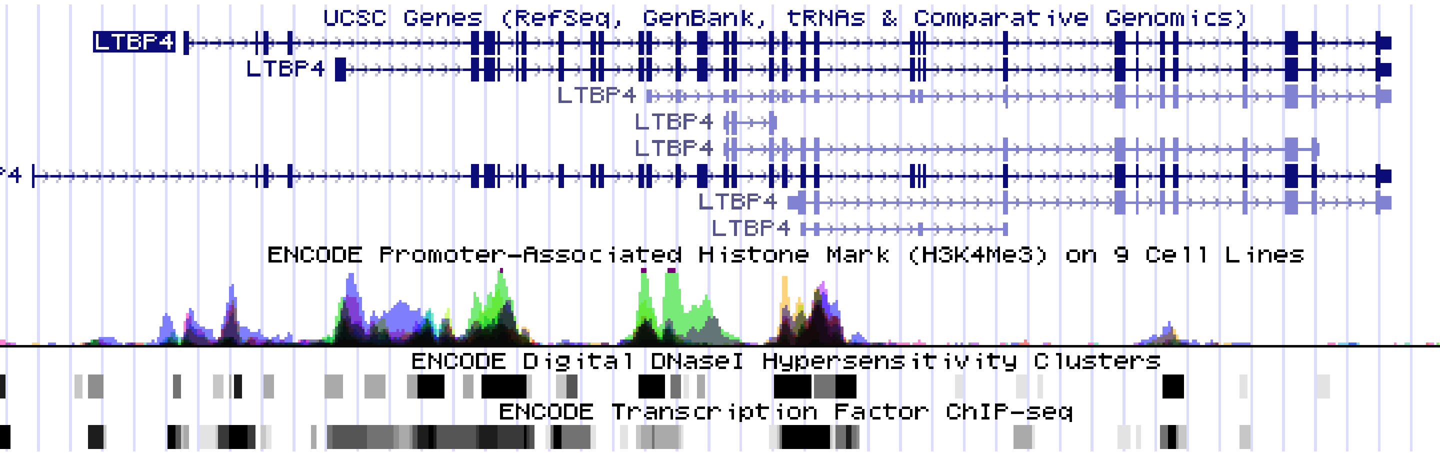

LTBP4 (continued)

C. Enlargement region around strongest *LTBP4* promoter

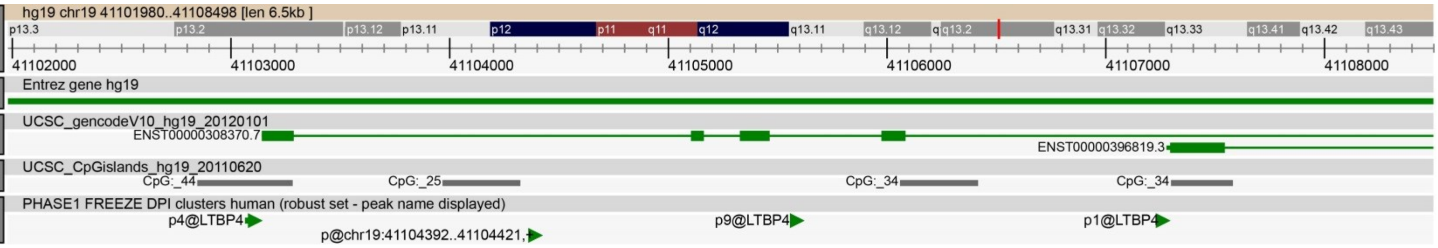

D. Enlargement of secondary *LTBP3* promoter region

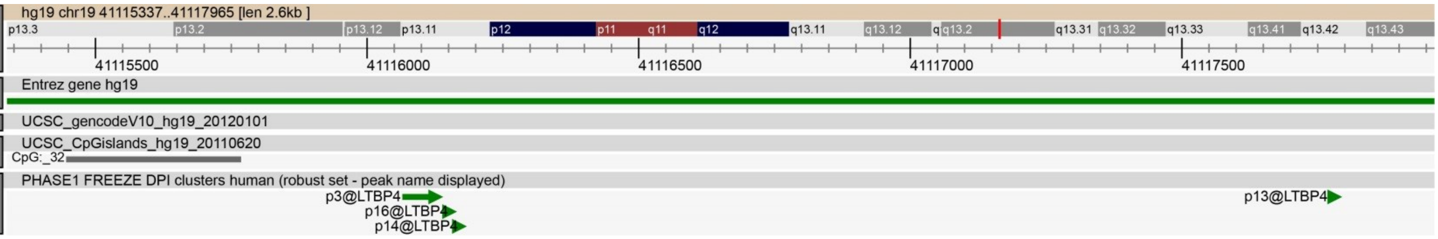

E. Enlargement of secondary *LTBP3* promoter region

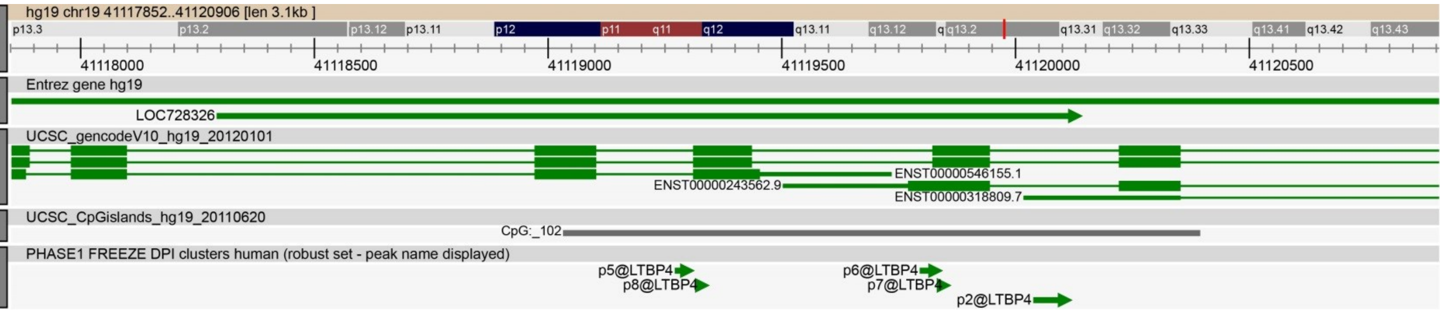

Supplement: Supplementary Figure — Promoters of the fibrillin/LTBP family members. [file mmc1.pdf]
