# Supplementary material for: Transcriptional profiling of the human fibrillin/LTBP gene family, key regulators of mesenchymal cell functions
Source: Mol Genet Metab. 2014 May;112(1):73–83. doi: 10.1016/j.ymgme.2013.12.006 (PMC4019825; doi:10.1016/j.ymgme.2013.12.006)
Supplement: Supplementary Table 1 — qPCR primers and antibodies. [file mmc2.pdf]

Gene Target Name	Primer	Sequence 5’-3’
Smooth Muscle Actin	ACTA2_fwd	AGCCAAGCACTGTCAGGAAT
	ACTA2_rvs	TTGTCACACACCAAGGCAGT
Biglycan	Bgn _fwd	CCCAGACCTCAAGCTCCTC
	Bgn_ rvs	TGGGACAGAAGTCGTTGACA
Collagen1a1	Col1a1_ fwd	GCCTGGTCAGAGAGGAGAGA
	Col1a1_ rvs	CTCCAGAGGGACCTTGTTTG
Collagen1a2	Col1a2_ fwd	AACACGTCTGGCTAGGAGAAAC
	Col1a2_ rvs	TTTCCTTGGAAGTCACTCCTTC
Fibrillin1	FBN1_fwd	GCTCCCAAACCCTGCAATTT
	FBN1_rvs	GGCAGTTGTGTTGCTTGGTTG
Fibrillin2	FBN2_fwd	TGTGCTGAAGGGTTACACGA
	FBN2_rvs	TCACAGATTCCTGGCTTGGT
Fibrillin3	FBN3_fwd	ACCTGGACGAATGCACCTC
	FBN3_rvs	CTGAGCTGACCAGGGTGAAG
Latent-transforming growth factor beta-binding protein 1	LTBP1_fwd	GCCCTAATGGTGAGTGTTTGA
	LTBP1_rvs	AGATCACAGGGGGATCAGG
Latent-transforming growth factor beta-binding protein 2	LTBP2_fwd	TGCCCTAGTGGAAAAGGCTA
	LTBP2_rvs	TCACACACTCATCCGCATCT
Latent-transforming growth factor beta-binding protein 3	LTBP3_fwd	CACCTGAGGACACAGAGGAAG
	LTBP3_rvs	GAGATCAGCTCGGGGTAGG
Latent-transforming growth factor beta-binding protein 4	LTBP4_fwd	CGTCAGGCCACCTACACAG
	LTBP4_rvs	CAGGGCCTCGAAGTCATCT

Protein Name	Primary Antibody (1:100)	Secondary Antibody (1:1000)
Fibrillin-1	Mouse monoclonal antibody to bovine fibrillin1 (ab3090_abcam)	Alexa Fluor 488 goat anti-mouse IgG (Invitrogen)
Fibrillin-2	Mouse monoclonal antibody to recombinant human Fibrillin2 (clone 48; Millipore)	Alexa Fluor 488 Goat anti-Mouse IgG (Invitrogen)
Smooth Muscle Actin	Rat monoclonal antibody to smooth muscle cells (ab15684_abcam)	Goat polyclonal to rat IgG-H&L Texas Red (ab6843_abcam)
Biglycan	Rabit polyclonal antibody to human Biglycan (ab49701_abcam)	Alexa Fluor 568 goat anti-rabbit IgG (Invitrogen)
Collagen1	Goat polyclonal antibody to human Collagen 1 (ab19811_abcam)	Alexa Fluor 633 donkey anti-goat IgG (Invitrogen)
